# Phylogenetically Widespread Polyembryony in Cyclostome Bryozoans and the Protracted Asynchronous Release of Clonal Brood-Mates

**DOI:** 10.1371/journal.pone.0170010

**Published:** 2017-01-17

**Authors:** Helen L. Jenkins, Andrea Waeschenbach, Beth Okamura, Roger N. Hughes, John D. D. Bishop

**Affiliations:** 1 Marine Biological Association of the United Kingdom, Plymouth, Devon, United Kingdom; 2 Department of Life Sciences, Natural History Museum, London, United Kingdom; 3 School of Biological Sciences, Bangor University, Bangor, Gwynedd, United Kingdom; NORWAY

## Abstract

Polyembryony–the production of multiple cloned embryos from a single fertilised egg–is a seemingly paradoxical combination of reproductive modes that nevertheless persists in diverse taxa. We document features of polyembryony in the Cyclostomata (Bryozoa)–an ancient order of modular colonial marine invertebrates–that suggest a substantial reduction in the paradoxical nature of this enigmatic reproductive mode. Firstly, we provide molecular evidence for polyembryony in three exemplar species, supporting the widely cited inference that polyembryony characterises the entire order. Secondly, genotyping demonstrates protracted release of cloned offspring from the primary embryo in a given gonozooid (chamber for embryonic incubation), thus exposing the same genotype to changing environmental conditions over time. Finally, we confirm that each gonozooid produces a distinct genotype, with each primary embryo being the result of a separate fertilisation event. We hypothesise that the sustained release of one or a few genotypes against varying environmental conditions achieves levels of risk-spreading similar to those in organisms that release multiple, unique genotypes at a single time. We argue that polyembryony, specifically with the production of a large number of progeny per fertilisation event, has been favoured in the Cyclostomata over long geological periods.

## Introduction

The apparent paradoxical nature of polyembryonic reproduction, where a sexually produced embryo divides into multiple genetically identical progeny, has long puzzled evolutionary biologists. Why should a single unproven genotype be replicated into numerous genetically identical propagules? George C. Williams [1] likened this process to purchasing multiple lottery tickets of the same number with no reason to favour one number over another. By combining cloning and sexual reproduction, the respective benefits of these contrasting reproductive modes appear compromised [2]. Nevertheless, polyembryony has evolved and persisted in a diverse range of taxa including rust fungi [3], algae [4], and animals (in [5]). In the Metazoa alone, more than 18 instances of polyembryony have been reported in ten classes across six phyla (Cnidaria (Hydrozoa), Platyhelminthes (Monogenea, Trematoda, Cestoda), Arthropoda (Cirripedia, Insecta), Bryozoa (Stenolaemata), Echinodermata (Asteroidea, Ophiuroidea), Chordata (Mammalia); in [5]).

Scenarios that are considered to favour the evolution of polyembryony include those where developing offspring have more information about their own environmental conditions than their mother had when the brood was produced. For example, in some parasites and parasitoids, clutch size may be adjusted after oviposition by asexual replication depending on host quality [5]. Thus, two-thirds of the reported metazoan examples of polyembryony are found in parasitic taxa. Similarly, in non-parasitic taxa polyembryonic cloning might allow rapid strategic adjustment of clutch size depending on food availability [5]. Polyembryony might also enhance fecundity in cases where sperm are in limited supply [6].

**Fig 1:**
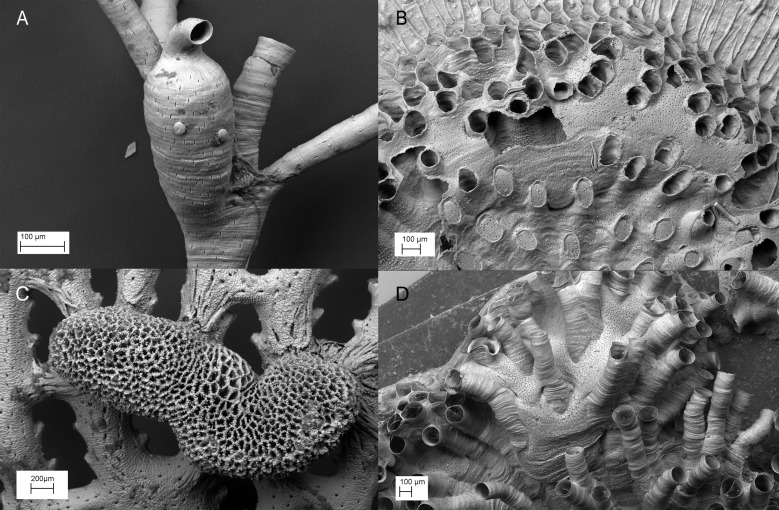
Images of exemplar of cyclostome bryozoan incubation chambers (gonozooids) from scanning electron microscopy. (A) *Filicrisia geniculata*, (B) *Plagioecia patina*, (C) *Hornera robusta* and (D) *Tubulipora plumosa*.

Within the Bryozoa, a phylum of modular colonial aquatic invertebrates, polyembryony is thought to occur throughout the entire order Cyclostomata (Class: Stenolaemata). Of the five stenolaemate orders that predominated throughout the Palaeozoic, cyclostomes were the only order surviving into the Jurassic following the Late Permian and Late Triassic mass extinction events [7]. Their generic diversity peaked in the Late Cretaceous but declined sharply following the Cretaceous-Paleogene boundary. Despite intense competition from members of the radiating order Cheilostomata, cyclostomes managed to maintain levels of diversity throughout the Cenozoic similar to those in the Early Cretaceous (see [8]). Although cheilostomes represent the great majority of bryozoan species diversity in the present day (>80%), cyclostomes have nevertheless persisted (contributing <10%) [9]. Their success has partly been attributed to their ability to incubate developing embryos, a trait also thought to have played a part in the radiation of cheilostomes (see [10]). Cyclostomes construct voluminous incubation chambers (gonozooids; Fig 1), in which up to 150 larvae per brood [11] arise from a small oligolecithal egg via embryonic cloning supported by matrotrophy [12]. The presence of gonozooids has been taken as indicative of polyembryony [13, 14], and the development of gonozooids in late Triassic cyclostomes [15, 16] suggests that polyembryony may be plesiomorphic for most post-Palaeozoic cyclostome bryozoans [13, 17]. The inference that polyembryony is widespread in cyclostomes is supported by early histological studies describing embryonic fission (the division of a primary embryo) in five cyclostome suborders [12, 18–22] across the major clades as identified by our most recent understanding of cyclostome phylogeny [23]. However, modern techniques have scarcely been used to confirm these early observations, or to exclude the possibility that the primary embryo is asexually derived (i.e. by apomictic parthenogenesis, although sperm production has been documented [12]) or that multiple fertilisation events occur within gonozooids. Thus, while recent genotyping analyses have confirmed polyembryony and distinct brood genotypes in *Crisia denticulata* [2, 24], further evidence is required to corroborate and characterise patterns of polyembryony across the order.

As sessile organisms, cyclostomes are at the mercy of the often highly variable abiotic and biotic conditions of benthic habitats, particularly in shallow waters. Thus, the question remains–why would a maternal colony (the mother) ‘bet’ on a single unproven offspring genotype at the expense of both (sexual) brood genetic diversity and her own relatively successful genotype [2, 5, 25]? Cyclostomes could circumvent the disadvantages of polyembryony if cloned offspring are released over prolonged periods of time from a given brood, often accompanied by the production of multiple broods of different genotype by the same colony [26]. By testing the suitability of genotypes against varying environmental conditions over time, polyembryony might achieve similar levels of risk-spreading as those attained by many sessile invertebrates that release a brood comprising multiple, unique genotypes at a single point in time. If so, polyembryony in cyclostome bryozoans may be less paradoxical than it appears at first glance. Preliminary evidence provides some support for these scenarios. Thus, it has been shown that broods within a single colony can be genetically distinct in at least one species [2]. Furthermore, illustrations by earlier authors show a range of developmental stages sharing the same gonozooid (generally a primary embryo in addition to secondary embryos and/or larvae) across three major clades (figured genera include: *Crisia* [12, 19], *Crisiella*, *Hornera*, and *Lichenopora* [12]). These observations are consistent with the continuous production of progeny within a gonozooid over a period of time, possibly from the same primary embryo. However, prolonged larval release from individual incubation chambers has not been documented. Nor is it known whether a succession of primary embryos, each derived from a separate fertilisation event and undergoing complete division to form a single, relatively brief, swarm of larvae, develops within a particular gonozooid.

In order to expand our understanding of polyembryony as a reproductive strategy, our study addresses the following outstanding questions regarding the Cyclostomata: 1) are larvae within incubation chambers truly polyembryonic throughout the order? 2) are cloned offspring released from individual gonozooids over extended periods of time? and 3) is it generally the case that each incubation chamber within a colony contains a distinct larval genotype? We use inter simple sequence repeat (ISSR) genotyping to document the sexual derivation and thus polyembryonic nature of cloned progeny in exemplar species representing the three major clades of Cyclostomata and microsatellite genotyping to characterise the prolonged production of larvae with the same genotype (and thus deriving from the same primary embryo) in the species *Filicrisia geniculata*. Resolving these questions enables us to propose a reduction in the apparent paradox of polyembryony for these sessile colonial animals.

## Materials and Methods

### Confirmation of polyembryony across the Cyclostomata

#### Taxon selection and tissue dissection

Chosen exemplar taxa representing the three major cyclostome clades (after [23]) were as follows: Clade A—*Plagioecia patina* (Family Plagioecidae; Gullmar fjord, Kristineberg, Sweden), Clade B—*Hornera robusta* (Family Horneridae; Shag Point, Otago, New Zealand), and Clade C—*Tubulipora plumosa* (Family Tubuliporidae; Hannafore Point, Looe, Cornwall, UK; Hoe Foreshore, Plymouth, Devon, UK). Polyembryony had been confirmed previously in *C*. *denticulata* (Family Crisiidae; Clade C) using microsatellite loci [2] thus, material of this species from Wembury, Devon (UK) was included here to verify ISSRs as reliable alternative markers. None of the species involved in this study are endangered or protected species. Collection permission was not required for UK taxa. Collecting in Sweden was carried out under the auspices of the laboratory operations and protocols of the Sven Loven Marine Centre (Göteborg University). In New Zealand, collections were made under a NZ Ministry for Primary Industries Special Permit (no. 464).

Larvae were collected from both live and RNAlater-preserved material. Individual gonozooids were isolated from colonies. Care was taken to avoid contamination with maternal tissue and only well-differentiated and clearly distinct individual larvae were collected. Larvae were rinsed in a drop of RNAlater, transferred into 5μl RNAlater, and stored at -20°C until DNA extraction. All available larvae were collected per gonozooid (see Table 1 for number collected). Although effort was made to dissect multiple incubation chambers per colony, this was not always possible due to the close proximity of gonozooids to one another (e.g. in some *T*. *plumosa* and *P*. *patina* colonies), in which case only a single incubation chamber per colony was dissected. When individual larvae could not be collected (e.g. from ethanol-preserved samples, in which larval tissue clumped together), whole-brood tissue samples (Table 1) were collected and stored in 10μl RNAlater at -20°C.

**Table 1:** ISSR brood screening information: details of species, broods examined, number of larvae screened per brood and ISSR markers used.

Species	Colony	Brood ID	No. of larvae	ISSR markers
*Crisia denticulata*	1	A	7	UBC 827, UBC 850, UBC 884
	2	B	5	
	3	C	6	UBC 827, UBC 850
*Hornera robusta*	1	D	10	UBC 817, UBC 855
	2	E	10	
	2	F	10	
*Plagioecia patina*	1	G	10	UBC 827, UBC 850, UBC 855
	2	H	9	
	2	WB 01*	N/A	
	3	I	10	
*Tubulipora plumosa*	1	J	6	UBC 817, UBC 855
	2	K	12	
	3	L	10	UBC 817, UBC 850, UBC 855
	4	M	8	
	4	WB 02	N/A	

Identical numbers for ‘Colony’ indicate broods that originated from the same colony. WB = whole-brood tissue sample.

* PCRs were too weak for automated electrophoresis.

#### DNA extraction, ISSR marker selection and amplification, and product visualization

For ISSR marker selection, gDNA was extracted from whole-brood samples using the DNeasy Blood & Tissue extraction (Qiagen) kit, following manufacturer’s instructions (= unmodified protocol). Total gDNA was extracted from individual RNAlater-preserved larvae following the modified DNeasy (Qiagen) animal tissue extraction protocol optimised for working with very small samples as detailed in Webster [27].

ISSR genotyping has been used to distinguish multi-locus genotypes (for details, see [28]) in botanical (e.g. [29, 30]) and zoological studies (e.g. [31, 32]). The relatively low per-species development costs make this method particularly advantageous for conducting multi-species investigations. However, because ISSR uses non-specific primers, particular care is needed with this technique to prevent the amplification of non-target DNA. Maternal genotypes were not used here because of the anticipated difficulty in completely separating clean maternal autozooidal tissue from internal and external surfaces bearing possible contaminants such as food particles, bacteria and microscopic epibionts. Incubated larvae are well protected from the outside world by the epithelial lining of the maternal body wall and mesothelial placental cells that directly surround the developing brood, and the calcified walls of the gonozooid itself, and thus expected to be contaminant-free. Examination of polyembryony using ISSR was therefore conducted by comparing the genotypes of single larvae (or ‘whole-brood’ tissue samples incorporating primary and secondary embryos and larvae).

Whole-brood gDNA extracts were used to select and optimise a subset of five ISSR markers (S1 Table) from a set of 20 primers originally developed for botanical studies (http://www.biosci.ohio-state.edu/~awolfe/ISSR/protocols.ISSR.html; see [33]) but also applied to metazoan taxa (e.g. [34]). Because not all five markers performed equally well for each species, the most informative 2–3 markers per species were chosen for subsequent brood screening. All selected markers gave reliable, polymorphic banding patterns differing consistently between species and indicating a degree of polymorphism between broods within the same species. Additionally, comparisons of the ISSR genotypes of single larvae from broods from different colonies were made to further demonstrate marker variability and the ability to resolve differences between broods.

PCRs were conducted in 25μl reaction volumes using Thermoprime or DreamTaq kits, 1μl of 10μM of a single ISSR primer and up to 12ng gDNA. PCR cycling conditions were as follows: initial denaturation for 3 min at 94°C, followed by 50 cycles of 30 s at 94°C, 30 s at T_a_°C (see S1 Table), 2 min at 72°C, and completed by 10 min at 72°C; the comparatively large number of PCR cycles was conducted to compensate for often low gDNA concentrations and limited amount of gDNA elute. For each larva and whole-brood gDNA extract, PCRs were conducted in triplicate to verify repeatability of results. Positive and negative controls were included. PCR success was checked by visualization on 0.8% agarose electrophoresis gels. Successful PCR products were purified using Millipore filter plates (Merck Millipore; processed by NHM Sequencing Facility).

High-resolution visualisation of PCR products was achieved by polyacrylamide gel electrophoresis (PAGE). 19% polyacrylamide gel mixture (38ml) was degassed for 3 mins prior to the addition of 32.5μl TEMED and 250μl 10% ammonium persulphate, set into 18 x 16 cm gels. Gels were run using a Hoefer SE600 cooled vertical electrophoresis apparatus at 150v for 8–9h. Gels were silver-stained using a rocking platform as follows: 2 x 3 min in Solution A (360ml distilled water, 40ml ethanol, 2ml acetic acid), 1 x 10 min in Solution B (200ml distilled water, 0.2g silver nitrate), 2 x 3 min in distilled water, 1 x 10 min in Solution C (300ml distilled water, 4.5g sodium hydroxide, 0.03g sodium borohydride, 1.2ml formaldehyde), 1 x 3 min in Solution A.

PCR products were selected for further analysis using an Experion automated electrophoresis station with the Experion DNA 1K kit (Biorad). This methodology obtains high-resolution virtual gel images from which band sizes are easily read, facilitating comparison and accurate scoring of banding patterns, particularly where closely spaced bands are present. Estimates of genetic divergence between broods were obtained by pairwise scoring of presence/absence of bands.

#### Brood screening protocol and ISSR profile scoring

Single-larva genotypes were compared as follows: within broods (*Comparison 1*) and between broods from the same colony (*Comparison 2*). Evidence of embryonic cloning is sought by *Comparison 1*, where an identical profile within a brood confirms larvae are clonemates derived from a single primary embryo that might be sexual or asexual. In order to rule out apomictic parthenogenesis, *Comparison 2* was carried out. Genotypes of broods produced by the same colony would be expected to be identical if they were the product of apomictic parthenogenesis. By demonstrating genotype variation between broods within colonies, evidence for reassortment, thus the sexual derivation of the primary embryo, and thus true polyembryony, is provided.

Each species was screened with 2–3 polymorphic ISSRs (see previous subsection; Table 1). Where replicate PCRs for a single larva produced different PAGE banding patterns, or PCRs failed (probably an indication of contamination or a problem with the PCR itself), larvae were discounted from the analysis. For each ISSR marker, unambiguous PCR products from three larvae per brood were chosen to be analysed using the Experion automated electrophoresis station. *Comparison 1* was carried out for all species. *Comparison 2*, using individual larvae, was conducted for *H*. *robusta*. *Comparison 2* using a combination of individual larvae and whole broods (Brood WB02 was divided into four samples) was conducted for *T*. *plumosa*. For *P*. *patina*, comparisons between Broods H and WB01 could not be made as PCR products for WB01 were too weak for automated electrophoresis (for PAGE gel, see Fig. H in S1 Appendix). Apomictic parthenogenesis had already been ruled out in *C*. *denticulata* by Hughes et al. [2] and was thus not included in *Comparison 2* analyses.

Experion banding patterns from larvae within the same brood were compared and band sizes verified. Bands were scored as the same locus when they were within the +/- 10% sizing accuracy limits specified by the manufacturer. From each of those bands, the average size was calculated and used in pairwise presence/absence comparisons between broods. Shared bands (between broods) were identified as such when they were within the +/- 10% sizing accuracy limits as specified by the manufacturer. Any bands difficult to differentiate between broods were scored as the same band (thus providing a conservative estimate of genetic variability), but could still be scored against a third brood devoid of that band. Only bands scored by Experion Software were used in the analysis.

### Sustained production of single-genotype larvae

#### Culturing and offspring capture

Culturing of *Filicrisia geniculata* was carried out in tanks filled with ~850ml of aged, 0.2μm-filtered, UV-sterilised natural seawater (FSW). Tanks were maintained at 16°C±1°C with 15:9 hour light:dark regime, and fed twice daily with a mixture of *Rhinomonas reticulata* and *Isochrysis galbana*. Water was replaced weekly and precautions were taken against any transfer of water-borne sperm between tanks [35].

Laboratory-controlled crosses between three colonies of *F*. *geniculata* (A-C) produced gonozooids that contained progeny of known parentage (Table 2). A maternal colony branch that produced a single, fully developed gonozooid was isolated, mounted onto a slide and placed in a separate acetate-lined tank. Tanks were inspected for the occurrence of metamorphosed larvae at approximately weekly intervals. Care was taken to record all metamorphosed larvae. Because unmetamorphosed larvae are hard to see and some were likely lost during water changes, the number of progeny recovered and analysed is likely to be an underestimate of the total output from a given gonozooid. Settled and metamorphosed larvae were allowed to grow into small colonies prior to preservation in 100% ethanol. For DNA extraction, a single branch, budded directly from the ancestrula, was dissected from each offspring colony.

**Table 2:** Protracted larval genotyping of *Filicrisia geniculata* broods.

Broods	Batch 1	Batch 2
Brood I (A x B)	21	4
Brood II (A x B)	13	3
Brood III (A x C)	16	11
Brood IV (A x C)	29	8

A, B and C indicate parental colonies that produced Broods I-IV. The number of progeny released from each brood during the first and second experimental period is shown as ‘Batch 1’ and ‘Batch 2’ respectively.

In order to prevent cross-fertilization between maternal colonies and maturing undetected offspring, the maternal colony branches were each placed into a new tank lined with clean acetate and cultured as above after an initial period of 30–35 days. Thus, progeny from the same gonozooid were collected in two successive batches over similar time intervals (30–35 days) (Table 2).

#### Microsatellite development

Tissue from fully developed *F*. *geniculata* gonozooids was used as the source of DNA for microsatellite development as it is free of food particles and epibionts (i.e. potential contaminants). Multiple (11–25) gonozooids per colony were sampled from a total of three colonies collected from Wembury, Devon, UK. gDNA extracts (for methodology, see ISSR section) were combined into a single preparation to capture intra-specific genetic diversity for optimal polymorphic primer design.

Polymorphic microsatellite markers were generated by GenoScreen (Lille, France) using the 454 GS-FLX platform (Roche Applied Science) [36]. Subsequent bioinformatic analysis of raw sequence data, performed using QDD software [37], identified sequences containing microsatellites and enabled the design of flanking PCR primer pairs. Of the total 28,177 raw sequence reads, 7,205 contained microsatellite motifs and 204 bioinformatically validated primer pairs were designed. From these, all microsatellites with > 9 motif repeats were selected in an attempt to obtain polymorphic loci [38]. This resulted in a total number of 34 loci, including di-, tri- and tetranucleotide microsatellite motifs (see S2 Table for full list).

A panel of eight individuals, which included the three brood parents involved in the controlled crosses (see previous subsection), was used to test the set of 34 primer pairs for PCR success and polymorphism. Total gDNA was extracted from clean, terminal branches using the unmodified protocol outlined in the previous (ISSR) section. PCRs were conducted in a total reaction volume of 20μl using GoTaq Flexi DNA Polymerase kit (Promega), 250nM unlabelled forward primer, 250nM unlabelled reverse primer, 1X buffer, 1.5mM MgCl_2_ solution, 250μM dNTP mix, 0.1mg/ml BSA, 0.5 units Go Taq DNA Polymerase and 2μl template gDNA. PCR cycling conditions were as follows: initial denaturation for 5 min at 95°C, followed by 35 cycles of 30 s at 95°C, 30 s at Ta°C (see S2 Table), 30 s at 72°C, and a final extension step of 10 min at 72°C. PCR products were visualised on 2% agarose gels. Loci that produced one or two bands per individual were further analysed using PAGE (see previous (ISSR) section; gels were stained with 10% SYBR Gold solution). Primer pairs that failed to amplify or that produced multiple fragments were discarded.

#### Microsatellite genotyping

Fragment analysis was conducted on all brood parents in order to test loci and establish parental genotypes. All progeny collected from a total of four broods, representing two successful crosses (see Table 2), were genotyped with three polymorphic loci, FG08, FG13 and FG17 (S1 Table), which were labelled with the fluorescent dyes PET, NED and 6-FAM, respectively. PCRs were performed as detailed in the previous subsection, but using 150nM labelled and 100nM unlabelled forward primer. PCRs were performed in simplex for each locus and combined for each individual to perform fragment analysis in multiplex. Fragment analysis was performed on an ABI 3130 Genetic Analyser (Applied Biosystems) and scored using Genemapper v 4.1 software (Applied Biosystems). Data were compiled and multilocus microsatellite genotypes were identified using the Multilocus Matches option in GenAlEx, ver. 6.3 [39, 40].

## Results

### Confirming polyembryony across the Cyclostomata using ISSRs

Comparisons of the genotypes of single larvae from broods from different colonies revealed differences in the presence/absence of ISSR bands in all pairwise comparisons within all examined species (Table 3; S3 Table; S2 Appendix), and demonstrates the ability of markers to distinguish between genotypes.

**Table 3:** Pairwise ISSR scores based on the brood scoring table (see S3 Table).

***Crisia denticulata***											
UBC 827				UBC 850				UBC 884			
	A	B	C		A	B	C		A	B	
A	-			A	-						
B	7	-		B	10	-		A	-		
C	2	7	-	C	10	12	-	B	5	-	
***Hornera robusta***											
UBC 817				UBC 855							
	D	E*	F*		D	E*	F*				
D	-			D	-						
E*	6	-		E*	10	-					
F*	4	6	-	F*	10	2	-				
***Plagioecia patina***											
UBC 827				UBC 850				UBC 855			
	G	H	I		G	H	I		G	H	I
G	-			G	-			G	-		
H	7	-		H	8	-		H	5	-	
I	5	6	-	I	3	5	-	I	8	3	-
***Tubulipora plumosa***											
UBC 817				UBC 855							
	J	K	L		J	K	L				
J	-			J	-						
K	6	-		K	3	-					
L	7	9	-	L	5	6	-				
UBC 817				UBC 850				UBC 855			
	M*	WB02*			M*	WB02*			M*	WB02*	
M*	-			M*	-			M*	-		
WB02*	7	-		WB02*	10	-		WB02*	4	-	

UBC numbers indicate primer identity. Numbers in cells indicate pairwise differences in banding patterns. Capital letters refer to brood identities; WB = whole brood.

*denotes pairwise comparisons between broods from the same colony.

Although an attempt was made to screen larvae from three different broods with three ISSR markers in both *C*. *denticulata* and *T*. *plumosa*, some broods were analysed using only two ISSRs [Brood C (UBC827 & UBC850), and Broods J and K (UBC817 & UBC855, respectively)] (Table 1). This was due to insufficient template DNA following failed PCR amplification or product degradation prior to automated electrophoresis.

*Comparison 1* revealed identical ISSR profiles (PAGE and virtual gel analysis; S1 Appendix & S2 Appendix) for all larvae within broods, thus providing evidence for embryonic cloning in all examined taxa.

*Comparison 2* was conducted on *H*. *robusta* and *T*. *plumosa* and revealed differences in banding profiles between broods from the same colony in both species, i.e. 6 and 2 different bands with ISSRs UBC817 and UBC855, respectively, between Broods E and F (*H*. *robusta*), and 7, 10 and 4 different bands with ISSRs UBC817, UBC850 and UBC855, respectively, between Broods M and WB02 (*T*. *plumosa*) (Table 3; S3 Table; Fig. B & Fig. E in S2 Appendix). These differences in genotypes between broods within colonies show that broods from different gonozooids represent different fertilisation events. Apomictic parthenogenesis is thus ruled out and this, in conjunction with evidence for embryonic cloning (Comparison 1), provides evidence for polyembryony in these taxa.

### Sustained production of single-genotype larvae in *Filicrisia geniculata*, using microsatellites

Genotyping analysis of the three microsatellite loci FG08, FG13 and FG17 revealed ten unique alleles to be distributed amongst the three parent colonies (A, B & C) and 101 progeny (Broods I-IV) (Table 4). Allele size ranged from 110–118 bp (in FG08), 186–238 bp (in FG13) and 192–228 bp (in FG17). FG17 was the most polymorphic locus with four alleles. Parental genotypes differed from each other at all three loci, except at locus FG08, where parents A and C had identical genotypes (114/114). Analysis of progeny and parental genotypes confirmed that the microsatellite loci were behaving as expected under the assumption of outcrossing, i.e. the offspring genotypes were composed of a mixture of alleles, one from each parent.

**Table 4:** Microsatellite genotypes for *Filicrisia geniculata* parents and progeny for loci FG08, FG13 and FG17.

	FG08		FG13		FG17	
**Cross A x B**						
A	114	114	194	238	228	228
B	110	118	186	238	222	228
Brood I progeny n = 25	110	114	186	238	228	228
Brood II progeny n = 15	110	114	186	194	222	228
**Cross A x C**						
A	114	114	194	238	228	228
C	114	114	186	194	192	218
Brood III progeny n = 26	114	114	194	238	192	228
Brood IV progeny n = 35	114	114	194	238	192	228
**N**_**t all**_	3		3		4	

Scores indicate allele size. Parents: A = father; B and C = mothers. Broods I & II and III & IV are replicate broods of Cross A x B and Cross A x C, respectively. N_t all_ = total no. of distinct alleles.

Progeny were obtained from single gonozooids of *F*. *geniculata* from two replicates each of crosses A x B (Broods I & II) and A x C (Broods III & IV) (Table 2). All broods continued to release larvae after the initial 30–35 day period. The longest period of larval release from a gonozooid was 69 days (Brood IV). Within each of Broods I-IV, all examined progeny shared an identical multilocus genotype (MLG) at all three loci, supporting the evidence for embryonic cloning obtained from ISSR analysis in other taxa (see previous section). The MLG differed between Broods I and II at loci FG13 and FG17, 186/238 and 186/194 and 228/228 and 222/228, respectively, but was identical at all loci for progeny of Brood III and IV (Table 4).

Progeny genotypes differed from the parental genotypes at loci FG08 (Brood I & II), FG13 (Brood II) and FG17 (Brood III & IV). Because of the ambiguous nature of one of the alleles (i.e. they were the same in mother and father), Brood I had the same genotype as the mother at locus FG13 (186/238) and the same as the father at locus FG17 (228/228), and Brood II had the same genotype as the mother at locus FG17 (222/228). Similarly, because locus FG13 allele 194 was present in both mother and father of cross A x C, Broods III and IV had the same genotype as the father at locus FG13 (194/238). Locus FG08, in this case, was entirely invariable, i.e. both parents and offspring had genotype 114/114 (Table 4).

In summary, microsatellite genotyping analysis indicated that: 1) MLG of offspring within a brood remained constant over time; 2) for each cross, the MLG of parents differed from each other and from that of their offspring; 3) a degree of genetic divergence between replicate broods was observed in one of the two crosses (A x B).

## Discussion

### Molecular evidence for polyembryony across divergent cyclostome lineages

This study provides the first genetic evidence for the occurrence of embryonic cloning in representatives of all the major cyclostome clades, i.e. *P*. *patina* (Clade A), *H*. *robusta* (Clade B), and *C*. *denticulata* and *T*. *plumosa* (Clade C) (clades from [23]). In addition, analyses of *H*. *robusta*, *T*. *plumosa* and *F*. *geniculata* demonstrated variation in genotypes between broods from the same colony (evidence for *F*. *geniculata* via cross A x B broods; Table 4). Although another *F*. *geniculata* cross (A x C) produced two broods of identical genotype (Table 4), this likely reflects a combination of sperm derived from the same father and low polymorphism in the microsatellite loci. Apomictic parthenogenesis, as a mechanism for producing embryos, can therefore be largely refuted in favour of embryonic cloning following the formation of a primary embryo by conventional sexual reproduction. We obtained evidence for outcrossing in *F*. *geniculata*, with two out of three microsatellite markers distinguishing progeny from the mother in all three distinct broods (Table 4). Selfing cannot strictly be ruled out in the taxa that were only examined using ISSRs.

Our evidence for polyembryony across all major clades of cyclostome bryozoans together with that from a previous study (*C*. *denticulata*: [2]) confirms early inferences of polyembryony based on microscopy [12, 18–22]. The molecular data demonstrate that polyembryony is associated with genetic variation both between maternal colonies and their offspring and between broods within colonies, and that this variation is generated by sexual reproduction. Combined with the near-ubiquity of large, voluminous gonozooids in cyclostome colonies and their occurrence since the late Triassic [15], this suggests that polyembryony likely characterises the entire order [13], with the possible exception of the family Cinctiporidae, for which no gonozooids have ever been recorded [41]. The large size of cinctiporid autozooids may enable them to contain multiple larvae generated by polyembryony [17] though histological studies are required to verify this hypothesis of intrazooidal incubation.

### Cyclostomes: an exceptional case of polyembryony

This study demonstrates that polyembryony in cyclostomes is characterised by a number of unique features. Firstly, cyclostomes are the only colonial metazoans that exhibit polyembryony. As a consequence of their modular architecture, cyclostome colonies are able to support more than one brood of offspring concurrently. Most cyclostomes produce one or a few gonozooids per colony ([42] p.271 and references therein), with the Crisiidae being somewhat unusual in often having multiple incubation chambers (e.g. [43]). Colonies of *F*. *geniculata*, *H*. *robusta*, *T*. *plumosa* (this study) and *C*. *denticulata* [2] have all been shown to produce multiple, genetically discrete broods. Thus, the capacity to form more than one incubation chamber by some of cyclostomes, each now shown to produce different larval genotypes in colonies representing the three major clades, reduces the apparent disparity between polyembryony and conventional sexual reproduction in this group. However, whether the convoluted and expansive gonozooids of the order Rectangulata (lichenoporids), which could facilitate the production of multiple primary embryos, contain only a single internal chamber and primary embryo, as suggested by Borg [44], remains to be examined.

Secondly, whilst viviparous embryonic incubation is not unique amongst polyembryonic metazoans (the other example being armadillos [45]), we show that offspring of the same genotype are released from a brood over an extended period of time, rather than all at once, in the cyclostome bryozoan *F*. *geniculata*. Observations of progeny at different stages of development within a dissected gonozooid in several taxa (*C*. *denticulata*: [43]; all taxa reported here, H Jenkins, pers. obs.) are consistent with these results and suggest the prolonged, iterative budding of young from the primary embryo. In the present study, the longest documented period of larval production was 69 days (Brood IV), a time period which is likely to encompass substantial changes in environmental conditions (e.g. in seasonal or other variations in food availability, temperature, competition, and/or predation pressure) experienced by the released larvae, ancestrulae and young colonies. Thus, the temporal environmental sampling by cloned offspring is a significant means of risk spreading—as indeed is implied by the large numbers of larvae produced within gonozooids (e.g. up to 150 [11]) relative to the number of gonozooids produced per colony (typically few [20]). Therefore, testing genotypes against varying environmental conditions over prolonged periods of time may enable crisiids and, by extension, other cyclostomes to avoid the limitations of polyembryony and may be especially important when only one or a few incubation chambers are produced.

Thirdly, as gonozooids are unable to feed, the production of broods and the prolonged release of larvae rely upon the transfer of nutrients from feeding zooids. This represents the division of labour between zooidal polymorphs within the cyclostome colony, allowing the sustained extra-embryonic nutrition of both primary and cloned embryos. Polyembryony in cyclostomes (via matrotrophy [46]) may therefore be characterised by maternal control of reproductive output, in terms of brood number and larval output, in response to resource availability. This scenario contrasts with polyembryony in nine-banded armadillos, which produce a single brood of a predetermined size (four embryos), possibly to circumvent constraints imposed by the single egg implantation site present in the uterus [45]. The cyclostome strategy is also unlike that of polyembryonic parasites and parasitoids, where cloning occurs after oviposition in host tissues, and where eventual brood size is, to an extent, influenced after oviposition by host quality [5].

Lastly, polyembryony is thought to occur throughout the entire order, or almost so. However, none of the ecological conditions suggested to favour polyembryony [5] are uniquely relevant to cyclostomes. For example, numerous non-polyembryonic organisms are likely to encounter fluctuations in food availability, and perhaps sperm limitation. Variation in host quality is irrelevant to non-parasitic organisms such as bryozoans,

It is possible that the inferred retention of polyembryony throughout the entire order Cyclostomata since at least the late Triassic [15] (an interval of approximately 200 My) could reflect a phylogenetic constraint [2]. However, it is difficult to envisage how polyembryony itself would impose such a constraint in cyclostomes. It has been suggested [43] that reduction in the degree of polyembryony could be readily achieved via a progressive decrease in the number of clonal progeny produced per primary embryo, with a compensatory increase in the number of gonozooids produced per colony, potentially leading to the complete loss of embryonic cloning within a given lineage. If polyembryony in cyclostomes were selectively neutral relative to sexual reproduction without embryonic cloning, random evolutionary walks would be expected over the 200 My time interval. We therefore should observe a continuum of forms ranging from those with one or a few gonozooids per colony, each producing numerous larvae, to forms with many, smaller, gonozooids per colony each producing one or a few larvae. The latter condition has, so far, not been recognised in cyclostomes. Typically, cyclostomes possess a few, large gonozooids (e.g. [20]), which are generally described as producing numerous larvae [12].

Polyembryony, specifically associated with the production of numerous progeny per primary embryo, therefore appears to have been conserved in cyclostomes over long geological periods and across a range of habitats, colony forms and population ecologies. One suitably pervasive feature of cyclostomes that may have contributed to the maintenance of polyembryony is the feeding apparatus (lophophore). Unlike in other bryozoans, the cyclostome lophophore does not fully extend into the surrounding water (but remains partially enclosed within the peristome), and the tentacles lack frontal cilia. Particle collection thus relies entirely on tentacle flicking to push particles into the central current [47]; this might possibly lead to inefficient capture of sperm from the water [43]; (see also [48]). Polyembryony could therefore be advantageous as a mechanism that yields numerous sexual progeny despite rare fertilisation events in cyclostomes [6]. If so, peculiarities of particle capture by the lophophore of cyclostomes could impose a phylogenetic constraint on reproductive mode within the group. However, it seems unlikely that sperm limitation is universal amongst cyclostomes. Pemberton *et al*. [43] failed to demonstrate a strong relationship between local population density of *C*. *denticulata*, as a proxy for sperm supply, and female reproductive success, as represented by the number of gonozooids per colony. Further investigation is required to determine the extent of sperm limitation in cyclostome populations.

The persistence of polyembryony amongst the Cyclostomata over geological time and into the present day demonstrates the success of this reproductive pattern [42] in the marine benthic environment. Nevertheless, cyclostomes have declined and remained diminished in diversity relative to the (non-polyembryonous) Cheilostomata since the Late Cretaceous (Campanian; [49], see also [8]). More clearly defining details of sexual reproduction across the order may improve our understanding of the maintenance of polyembryony and its limitations in cyclostomes. Future investigations, for example on the duration of larval release from primary embryos in additional species, the possibility of multiple primary embryos within gonozooids in certain taxa such as lichenoporids, the maternal control of larval production, the role of parent-offspring (and inter-brood) conflict over resources, and the influence of environmental variation on larval development and release, may enable deeper understanding of the drivers and maintenance of polyembryony.

In conclusion, the unique case of polyembryony in cyclostome bryozoans contributes to our wider understanding of the evolution and persistence of this enigmatic reproductive mode in metazoans. In particular, by providing evidence for protracted, asynchronous release of clonal brood-mates and the concurrent production of multiple genetically discrete broods, both processes that are facilitated by the polymorphic modular architecture of their colonies, cyclostomes reveal how the apparent costs of polyembryony might be substantially reduced.

## Supporting Information

S1 Table Primers used for ISSR and microsatellite genotyping. (DOCX)

S2 Table Details of the 34 microsatellite primer pairs tested for the genotyping analysis of *Filicrisia geniculata*. (DOCX)

S3 Table Overview of brood scores, as inferred from automated electrophoresis (Experion). (DOCX)

S1 Appendix ISSR Genotyping Analysis–PAGE gels. (PDF)

S2 Appendix ISSR Genotyping Analysis–virtual gels. (PDF)
