# Supplementary material for: Phylogenetically Widespread Polyembryony in Cyclostome Bryozoans and the Protracted Asynchronous Release of Clonal Brood-Mates
Source: PLoS One. 2017 Jan 17;12(1):e0170010. doi: 10.1371/journal.pone.0170010 (PMC5240946; doi:10.1371/journal.pone.0170010)
Supplement: S1 Table — (DOCX) [file pone.0170010.s001.docx]

**S1 Table.** **Primers used for ISSR and microsatellite genotyping.**

Ta = primer annealing temperature. A range = size range of alleles (bp)

| **Locus** | **Sequence (5’ – 3’)** | **T_a_ (°C)** | **Repeat motif** | **A range** |
| --- | --- | --- | --- | --- |
| **ISSRs** |  |  |  |  |
| UBC 817 | (CA)_8_A | 45 |  |  |
| UBC 827 | (AC)_8_G | 50 |  |  |
| UBC 850 | (GT)_8_YC | 50 | N/A | N/A |
| UBC 855 | (AC)_8_YT | 45 |  |  |
| UBC 884 | HBH(AG)_7_ | 35 |  |  |
| **Microsatellites** |  |  |  |  |
| FG08 | F: TCACCTCCTCATACACGCCT  R: TCTGTGCTGTATTGTGAGCG | 52 | (CAAA)_11_ | 110-118 |
| FG13 | F: ACATTAGACCCGGGATTTCG  R: AAGTTGTGAAGTTAAGTTGTTCCAA | 51 | (AC)_9_ | 186-238 |
| FG17 | F: TTTAAAATCCACACTCTATCGCC  R: CAGGTACACTTACATGCCAACTACA | 52 | (TGTA)_23_ | 176-228 |
