# Supplementary material for: Phylogenetically Widespread Polyembryony in Cyclostome Bryozoans and the Protracted Asynchronous Release of Clonal Brood-Mates
Source: PLoS One. 2017 Jan 17;12(1):e0170010. doi: 10.1371/journal.pone.0170010 (PMC5240946; doi:10.1371/journal.pone.0170010)

**S2 Table. Details of the 34 microsatellite primer pairs tested for the genotyping analysis of *Filicrisia geniculata*.** T_a_ (ºC), annealing temperature; N_cycl_ , no. of PCR cycles. The Amplification and profile test column indicates the level of analysis used to test the loci: all PCR products were screened on agarose gels, some were subsequently visualised on acrylamide gels, and some were tested using fragment analysis. Banding profiles indicate whether primers amplified a single locus or multiple loci. PCR product sizes were obtained from the primer analysis conducted by Genoscreen. Loci FG08, FG13 & FG17 were used in fragment analysis.


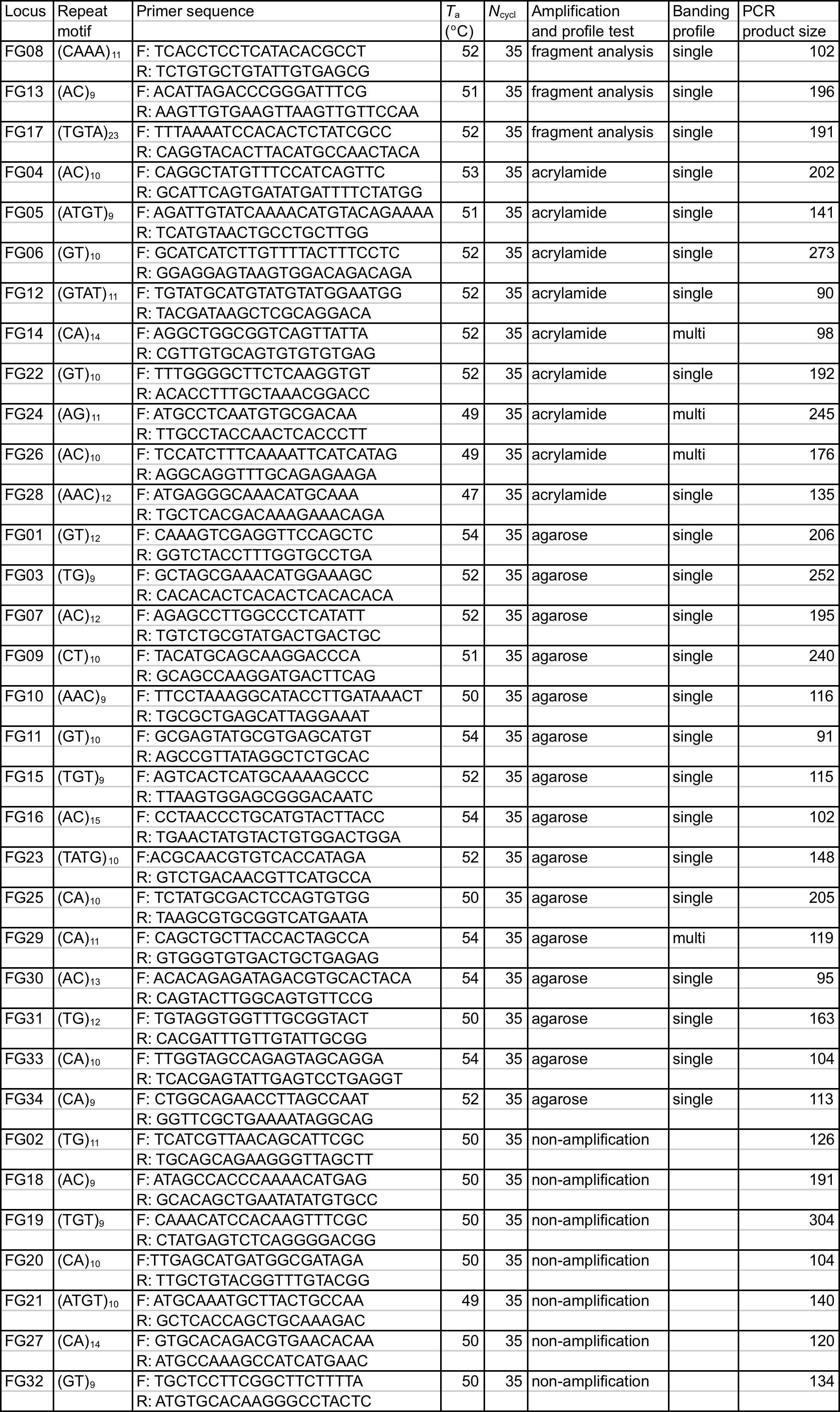

Supplement: S2 Table — (DOCX) [file pone.0170010.s002.docx]
