# Supplementary material for: Phylogenetically Widespread Polyembryony in Cyclostome Bryozoans and the Protracted Asynchronous Release of Clonal Brood-Mates
Source: PLoS One. 2017 Jan 17;12(1):e0170010. doi: 10.1371/journal.pone.0170010 (PMC5240946; doi:10.1371/journal.pone.0170010)
Supplement: S3 Table — (DOCX) [file pone.0170010.s003.docx]

**S3 Table. Overview of brood scores, as inferred from automated electrophoresis (Experion).**

(i) *Crisia denticulata*, (ii) *Hornera robusta*, (iii) *Plagioecia patina*, and (iv) *Tubulipora plumosa*. Grey cell = band present.

| (i) ***Crisia denticulata*** | | | | |
| --- | --- | --- | --- | --- |
|  | Band size (bp) | Brood A | Brood B | Brood C |
| UBC 827 | | | | |
| 1 | 720 |  |  |  |
| 2 | 751 |  |  |  |
| 3 | 820 |  |  |  |
| 4 | 843/862 |  |  |  |
| 5 | 970 |  |  |  |
| 6 | 1202 |  |  |  |
| 7 | 1252 |  |  |  |
| 8 | 1357 |  |  |  |
| UBC 850 | | | | |
| 1 | 312 |  |  |  |
| 2 | 451 |  |  |  |
| 3 | 468/470 |  |  |  |
| 4 | 490/492.5 |  |  |  |
| 5 | 575 |  |  |  |
| 6 | 583/585 |  |  |  |
| 7 | 652 |  |  |  |
| 8 | 670 |  |  |  |
| 9 | 691.5/699 |  |  |  |
| 10 | 814 |  |  |  |
| 11 | 825 |  |  |  |
| 12 | 857 |  |  |  |
| 13 | 946/951 |  |  |  |
| 14 | 990 |  |  |  |
| 15 | 1308 |  |  |  |
| 16 | 1351 |  |  |  |
| UBC 884 | | | | |
| 1 | 402/408 |  |  | n/a |
| 2 | 677/686 |  |  | n/a |
| 3 | 732.5 |  |  | n/a |
| 4 | 968 |  |  | n/a |
| 5 | 1002 |  |  | n/a |
| 6 | 1283 |  |  | n/a |
| 7 | 1327 |  |  | n/a |

| (ii) ***Hornera robusta*** | | | | |
| --- | --- | --- | --- | --- |
|  | Band size (bp) | Brood D | Brood E | Brood F |
| UBC 817 | | | | |
| 1 | 557 |  |  |  |
| 2 | 559/571 |  |  |  |
| 3 | 841 |  |  |  |
| 4 | 848/861 |  |  |  |
| 5 | 1038/1043 |  |  |  |
| 6 | 1087 |  |  |  |
| 7 | 1172 |  |  |  |
| 8 | 1212/1264 |  |  |  |
| UBC 855 | | | | |
| 1 | 644/645 |  |  |  |
| 2 | 654 |  |  |  |
| 3 | 726/728 |  |  |  |
| 4 | 749.5 |  |  |  |
| 5 | 858/862 |  |  |  |
| 6 | 882 |  |  |  |
| 7 | 981 |  |  |  |
| 8 | 1100/1105 |  |  |  |
| 9 | 1129 |  |  |  |
| 10 | 1267/1271 |  |  |  |
| 11 | 1372 |  |  |  |

| (iii) ***Plagioecia patina*** | | | | |
| --- | --- | --- | --- | --- |
|  | Band size (bp) | Brood G | Brood H | Brood I |
| UBC 827 | | | | |
| 1 | 448 |  |  |  |
| 2 | 677 |  |  |  |
| 3 | 701 |  |  |  |
| 4 | 824 |  |  |  |
| 5 | 871 |  |  |  |
| 6 | 993 |  |  |  |
| 7 | 1049.5 |  |  |  |
| 8 | 1097/1113 |  |  |  |
| 9 | 1164 |  |  |  |
| UBC 850 | | | | |
| 1 | 421/430 |  |  |  |
| 2 | 466 |  |  |  |
| 3 | 471/474 |  |  |  |
| 4 | 699/727.5 |  |  |  |
| 5 | 819 |  |  |  |
| 6 | 798/854 |  |  |  |
| 7 | 1086/1099 |  |  |  |
| 8 | 1192/1212 |  |  |  |
| UBC 855 | | | | |
| 1 | 563 |  |  |  |
| 2 | 634/638 |  |  |  |
| 3 | 660/668 |  |  |  |
| 4 | 679 |  |  |  |
| 5 | 733/740 |  |  |  |
| 6 | 765 |  |  |  |
| 7 | 855 |  |  |  |
| 8 | 885/902 |  |  |  |
| 9 | 1218/1256/1282 |  |  |  |

| (iv) ***Tubulipora plumosa*** | | | | |
| --- | --- | --- | --- | --- |
|  | Band size (bp) | Brood J | Brood K | Brood L |
| UBC 817 | | | | |
| 1 | 426 |  |  |  |
| 2 | 441 |  |  |  |
| 3 | 613 |  |  |  |
| 4 | 641 |  |  |  |
| 5 | 688 |  |  |  |
| 6 | 872 |  |  |  |
| 7 | 1013 |  |  |  |
| 8 | 1058.2 |  |  |  |
| 9 | 1240 |  |  |  |
| 10 | 1296 |  |  |  |
| 11 | 1341.5/1342 |  |  |  |
| UBC 855 | | | | |
| 1 | 354 |  |  |  |
| 2 | 874 |  |  |  |
| 3 | 983 |  |  |  |
| 4 | 1002 |  |  |  |
| 5 | 1165 |  |  |  |
| 6 | 1205.5 |  |  |  |
| 7 | 1302/1325 |  |  |  |
|  | Band size (bp) | Brood M | WB02 | - |
| UBC 817 | | | | |
| 1 | 461/463 |  |  | - |
| 2 | 477 |  |  | - |
| 3 | 678 |  |  | - |
| 4 | 781 |  |  | - |
| 5 | 879 |  |  | - |
| 6 | 906 |  |  | - |
| 7 | 1121 |  |  | - |
| 8 | 1205 |  |  | - |
| UBC 850 | | | | |
| 1 | 541 |  |  | - |
| 2 | 616 |  |  | - |
| 3 | 622 |  |  | - |
| 4 | 644.5 |  |  | - |
| 5 | 656 |  |  | - |
| 6 | 691 |  |  | - |
| 7 | 755/762 |  |  | - |
| 8 | 836 |  |  | - |
| 9 | 985.5/992 |  |  | - |
| 10 | 1115 |  |  |  |
| 11 | 1200 |  |  | - |
| 12 | 1294.5 |  |  | - |
| UBC 855 | | | | |
| 1 | 481 |  |  | - |
| 2 | 518 |  |  | - |
| 3 | 581 |  |  | - |
| 4 | 625/627.5 |  |  | - |
| 5 | 778.5 |  |  | - |
| 6 | 931/932 |  |  | - |
| 7 | 1056.5/1076 |  |  | - |
