# Supplementary material for: Phylogenetically Widespread Polyembryony in Cyclostome Bryozoans and the Protracted Asynchronous Release of Clonal Brood-Mates
Source: PLoS One. 2017 Jan 17;12(1):e0170010. doi: 10.1371/journal.pone.0170010 (PMC5240946; doi:10.1371/journal.pone.0170010)
Supplement: S1 Appendix — (PDF) [file pone.0170010.s004.pdf]

S1 Appendix. ISSR Genotyping Analysis – PAGE gels

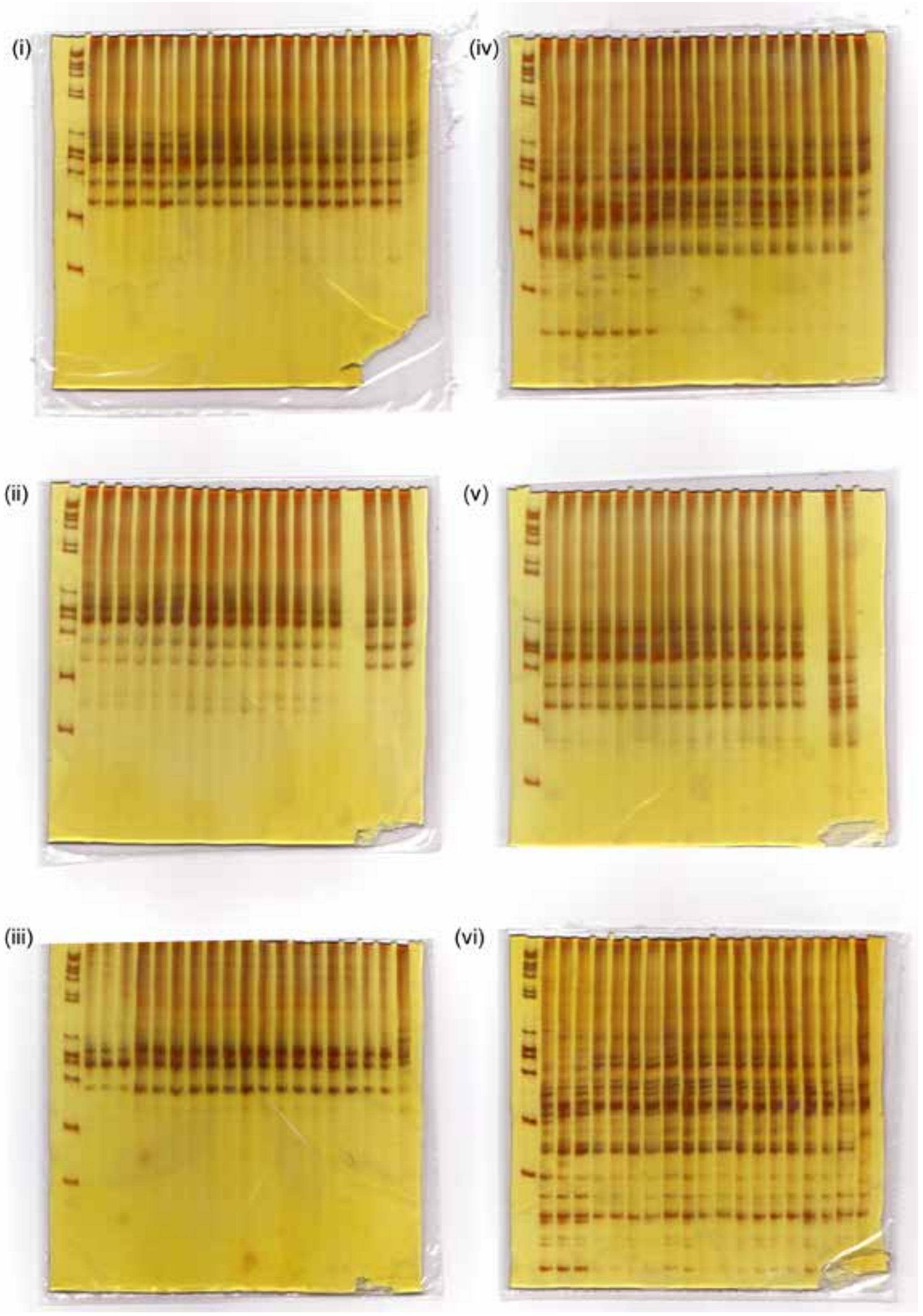

Figure A: PAGE gels from genotyping analysis of *Crisia denticulata*: within-brood comparison. Gel images show banding profiles of larvae (in triplicate) from each brood screened with the ISSR primers UBC 827 and UBC 850. UBC 827: (i) Brood A, (ii) Brood B & Brood A (lanes 18-20 only), (iii) Brood C. UBC 850: (iv) Brood A, (v) Brood B & Brood A (lanes 18-20 only), (vi) Brood C. Lane 1 = ladder. Note: Lane 17 in (ii) and (v) is intentionally left blank.

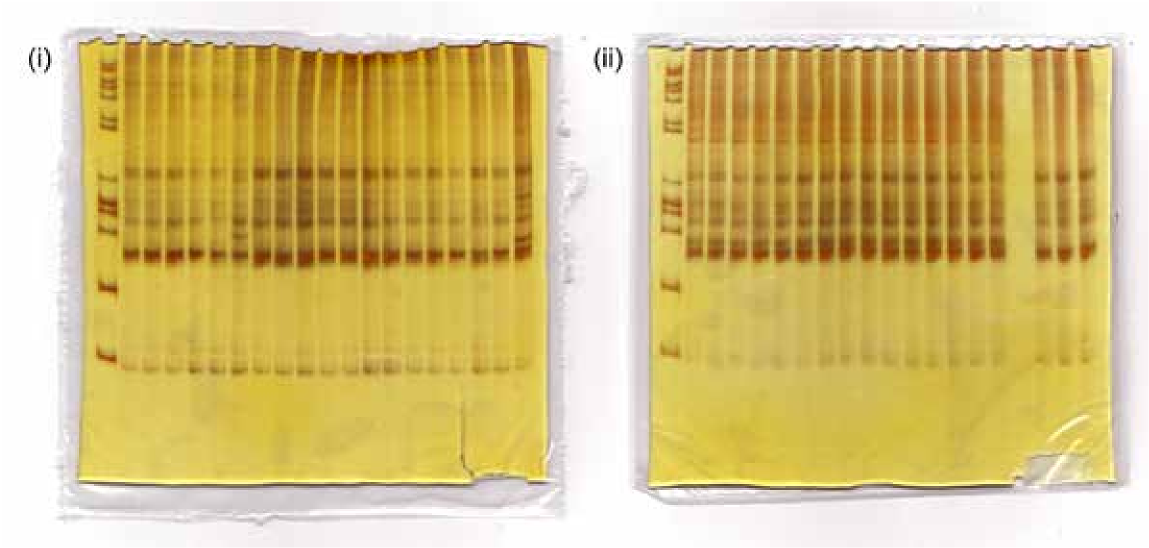

Figure B: PAGE gels from genotyping analysis of *Crisia denticulata*: within-brood comparison. Gel images show banding profiles of larvae (in triplicate) from each brood screened with the ISSR primer UBC 884: (i) Brood A, (ii) Brood B & Brood A (Lanes 18-20 only). Lane 1 = ladder. Note: Lane 17 in (ii) is intentionally left blank.

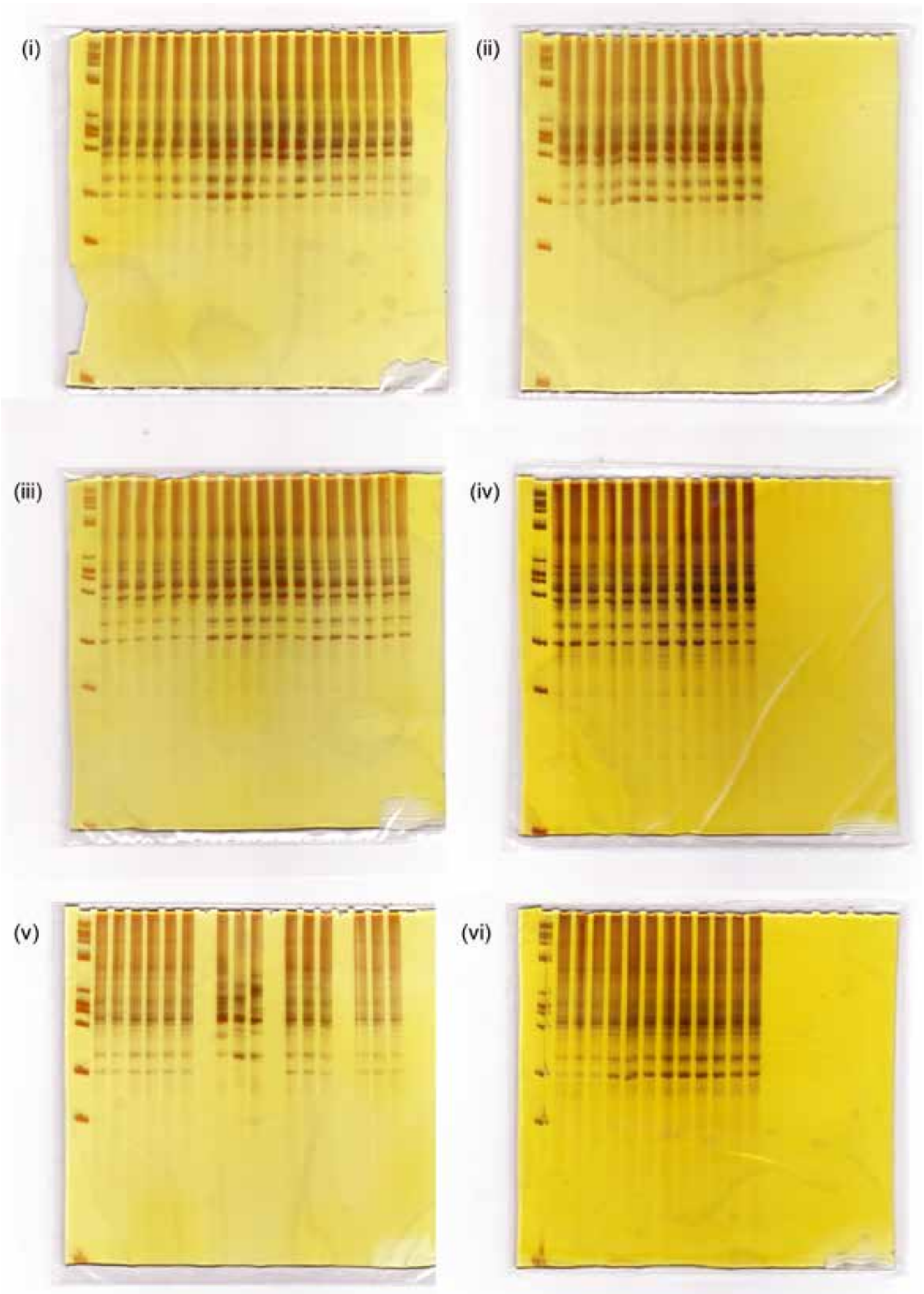

Figure C: PAGE gels from genotyping analysis of *Hornera robusta*: within-brood comparison. Gel images show banding profiles of larvae (in triplicate) from each brood screened with the ISSR primer UBC 817: (i-ii) Brood D, (iii-iv) Brood E, (v-vi) Brood F. Lane 1 = ladder. Note: Lanes 8, 12 & 16 in (v) are intentionally left blank.

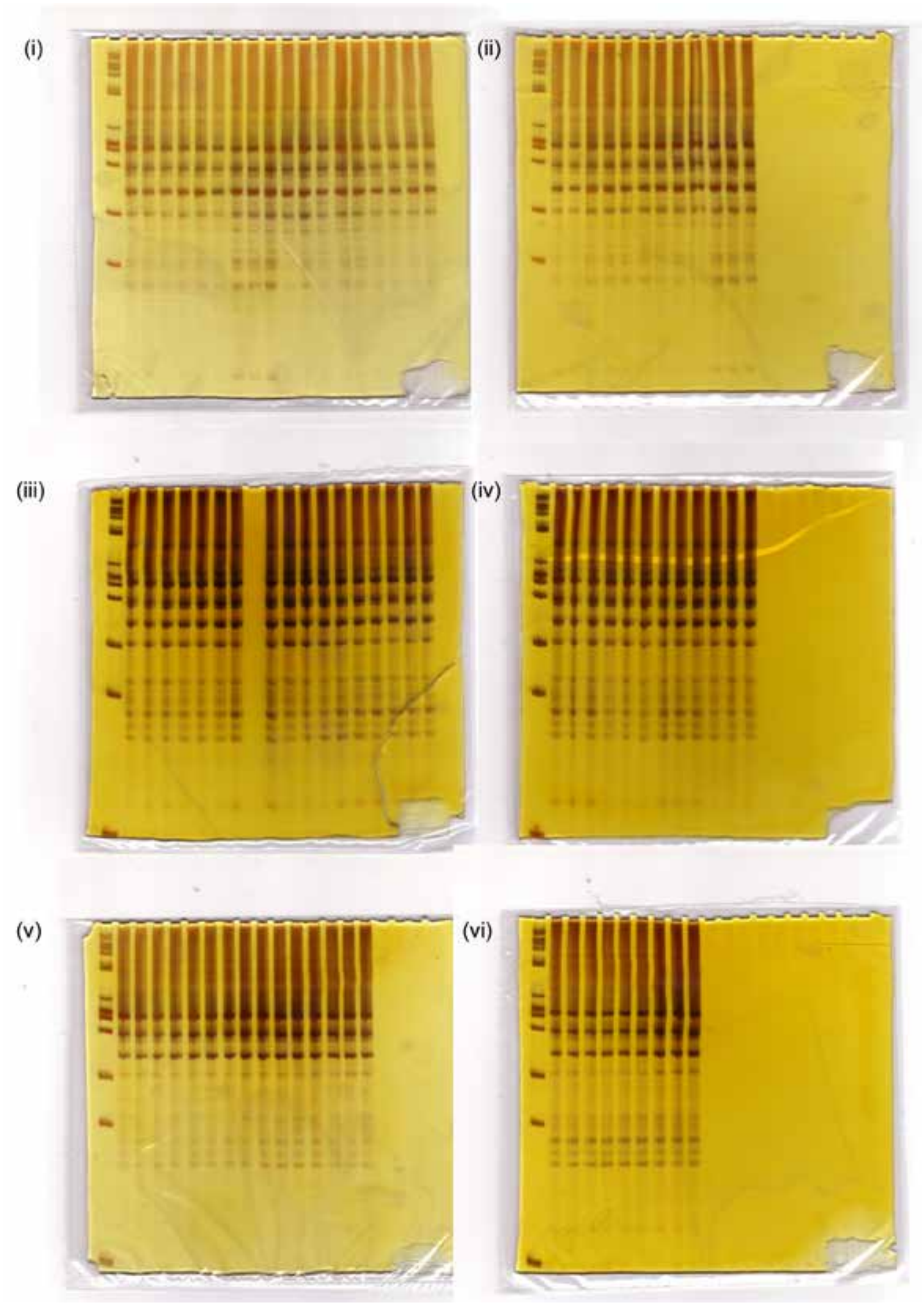

Figure D: PAGE gels from genotyping analysis of *Hornera robusta*: within-brood comparison. Gel images show banding profiles of larvae (in triplicate) from each brood screened with the ISSR primer UBC 855: (i-ii) Brood D, (iii-iv) Brood E, (v-vi) Brood F. Lane 1 = ladder. Note: Lane 9 in (iii) is blank due to PCR failure.

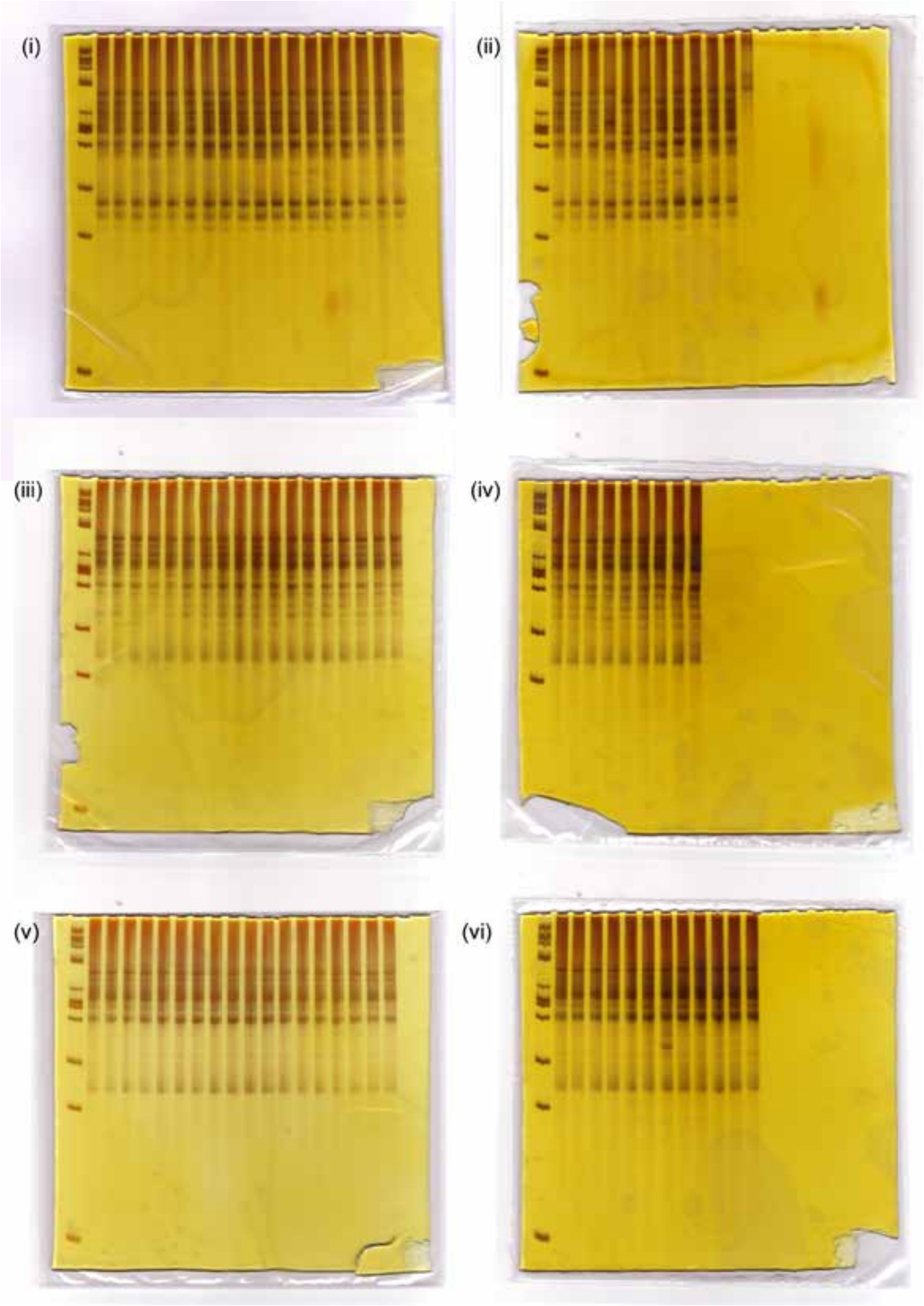

Figure E: PAGE gels from genotyping analysis of *Plagioecia patina*: within-brood comparison. Gel images show banding profiles of larvae (in triplicate) from each brood screened with the ISSR primer UBC 827: (i-ii) Brood G, (iii-iv) Brood H, (v-vi) Brood I. Lane 1 = ladder.

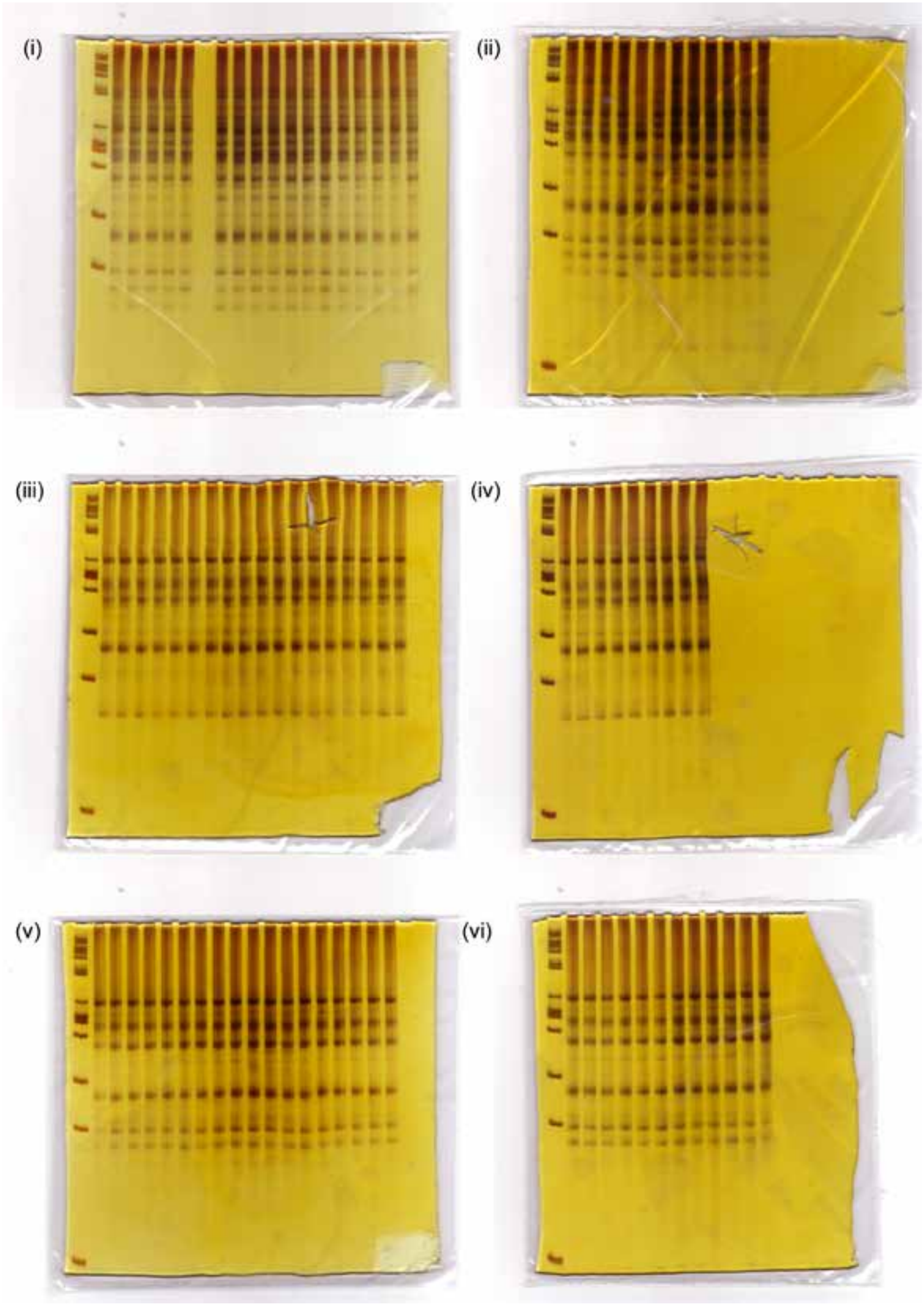

Figure F: PAGE gels from genotyping analysis of *Plagioecia patina*: within-brood comparison. Gel images show banding profiles of larvae (in triplicate) from each brood screened with the ISSR primer UBC 850: (i-ii) Brood G, (iii-iv) Brood H, (v-vi) Brood I. Lane 1 = ladder. Note: Lane 7 in (i) is intentionally left blank.

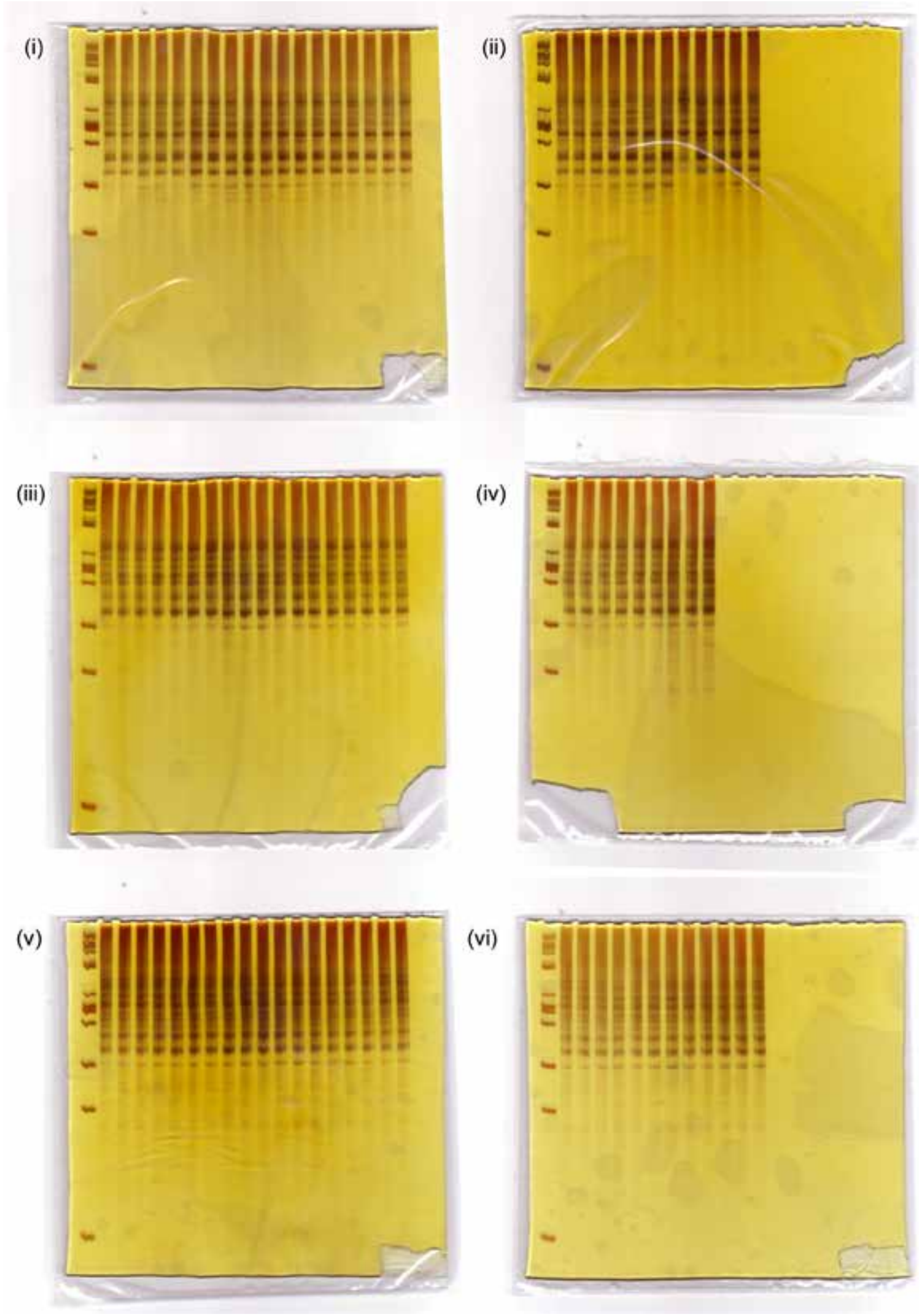

Figure G: PAGE gels from genotyping analysis of *Plagioecia patina*: within-brood comparison. Gel images show banding profiles of larvae (in triplicate) from each brood screened with the ISSR primer UBC 855: (i-ii) Brood G, (iii-iv) Brood H, (v-vi) Brood I. Lane 1 = ladder.

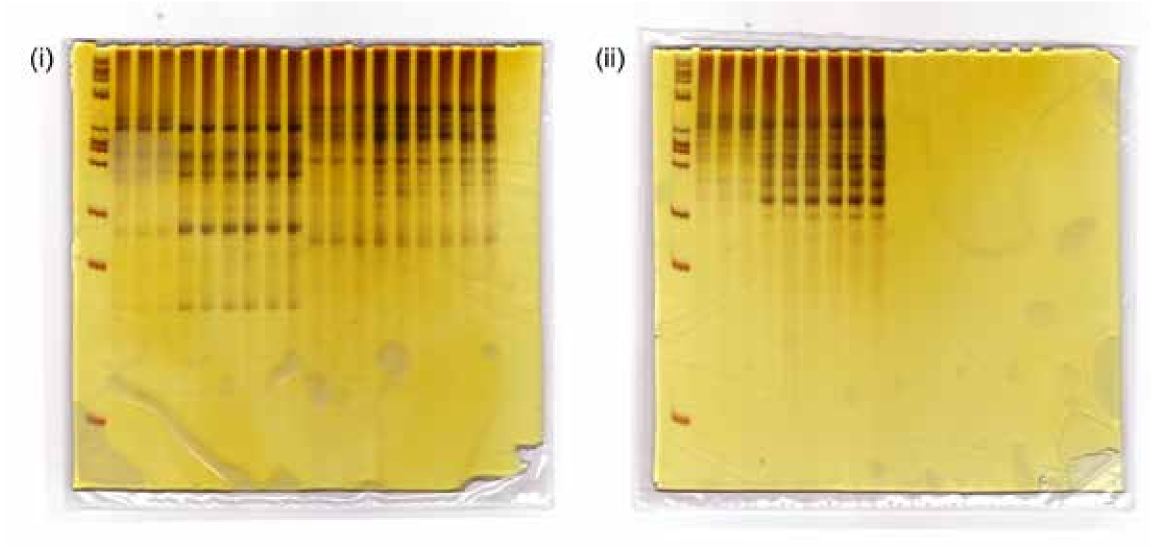

Figure H: PAGE gels from genotyping analysis of *Plagioecia patina*: within-brood comparison and comparison between broods within the same colony. Gel images show banding profiles of six Brood H larvae and 'whole brood' sample WB 01 (in triplicate) screened with the ISSR primers UBC 827, UBC 850 and 855: (i) UBC 850: WB 01 (lanes 2-4), Brood H (lanes 5-10); UBC 827: WB 01 (lanes 11-13), Brood H (lanes 14-19). (ii) UBC 855: WB 01 (lanes 2-4), Brood H (lanes 5-10). Lane 1 = ladder.

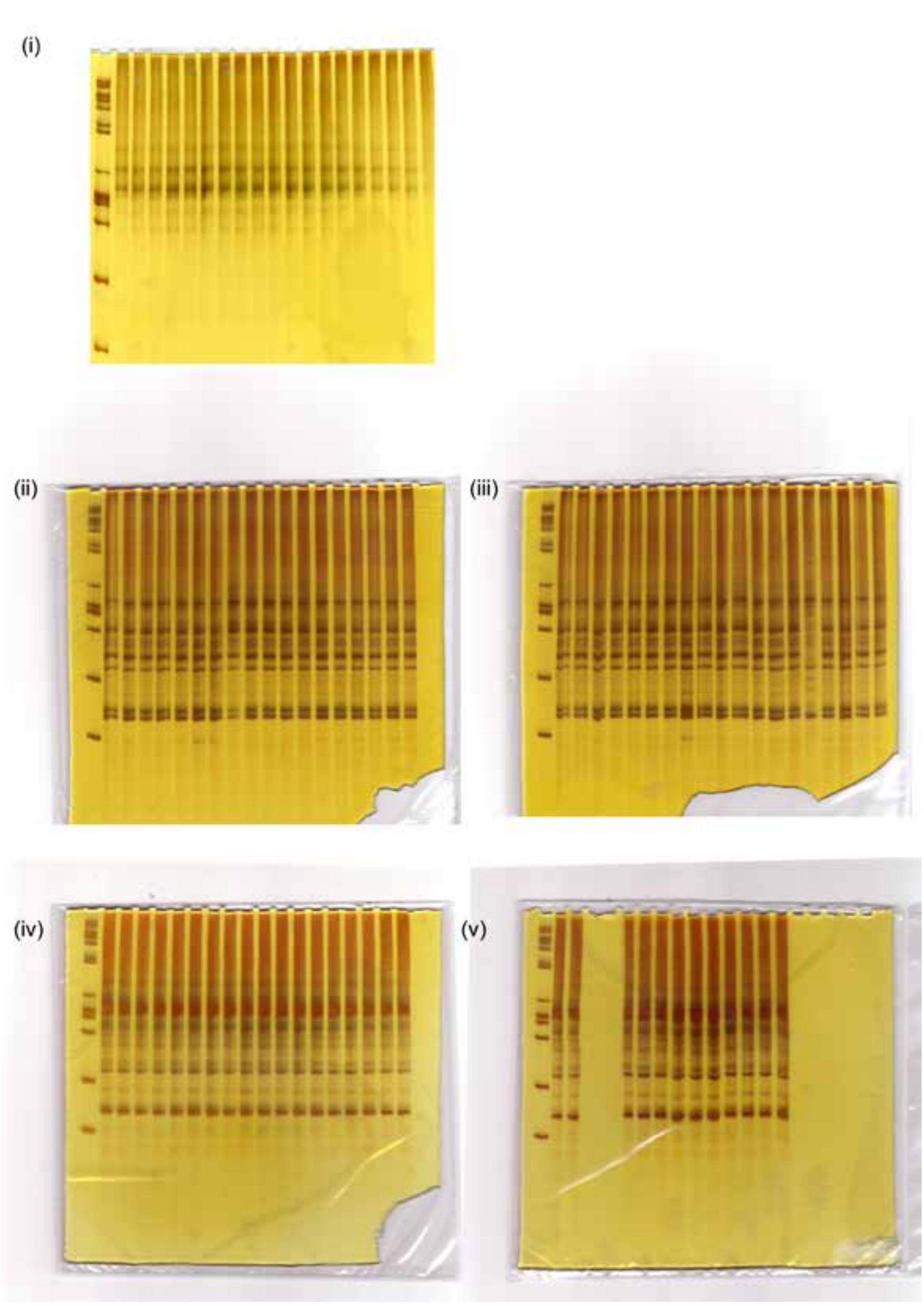

Figure I: PAGE gels from genotyping analysis of *Tubulipora plumosa*: within-brood comparison. Gel images show banding profiles of larvae (in triplicate) from each brood screened with the ISSR primer UBC 817: (i) Brood J, (ii-iii) Brood K, (iv-v) Brood L. Lane 1 = ladder. Note: Lanes 4 & 5 in (v) are intentionally left blank.

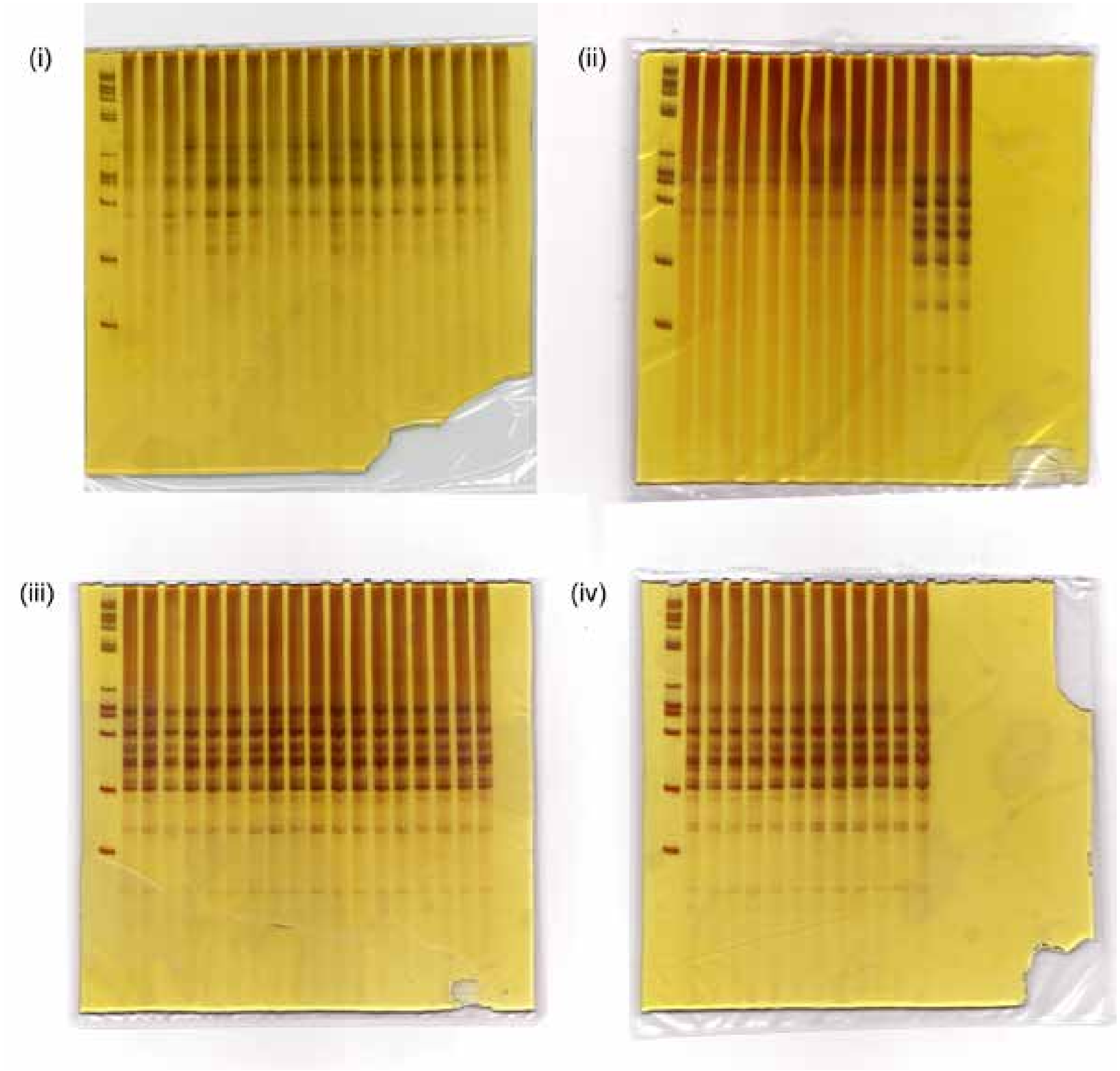

Figure J: PAGE gels from genotyping analysis of *Tubulipora plumosa*: within-brood comparison. Gel images show banding profiles of larvae (in triplicate) from each brood screened with the ISSR primer UBC 850: (i) Brood J, (ii) Broods J & L (lanes 2-12 & 13-15, respectively), (iii-iv) Brood L. Lane 1 = ladder.

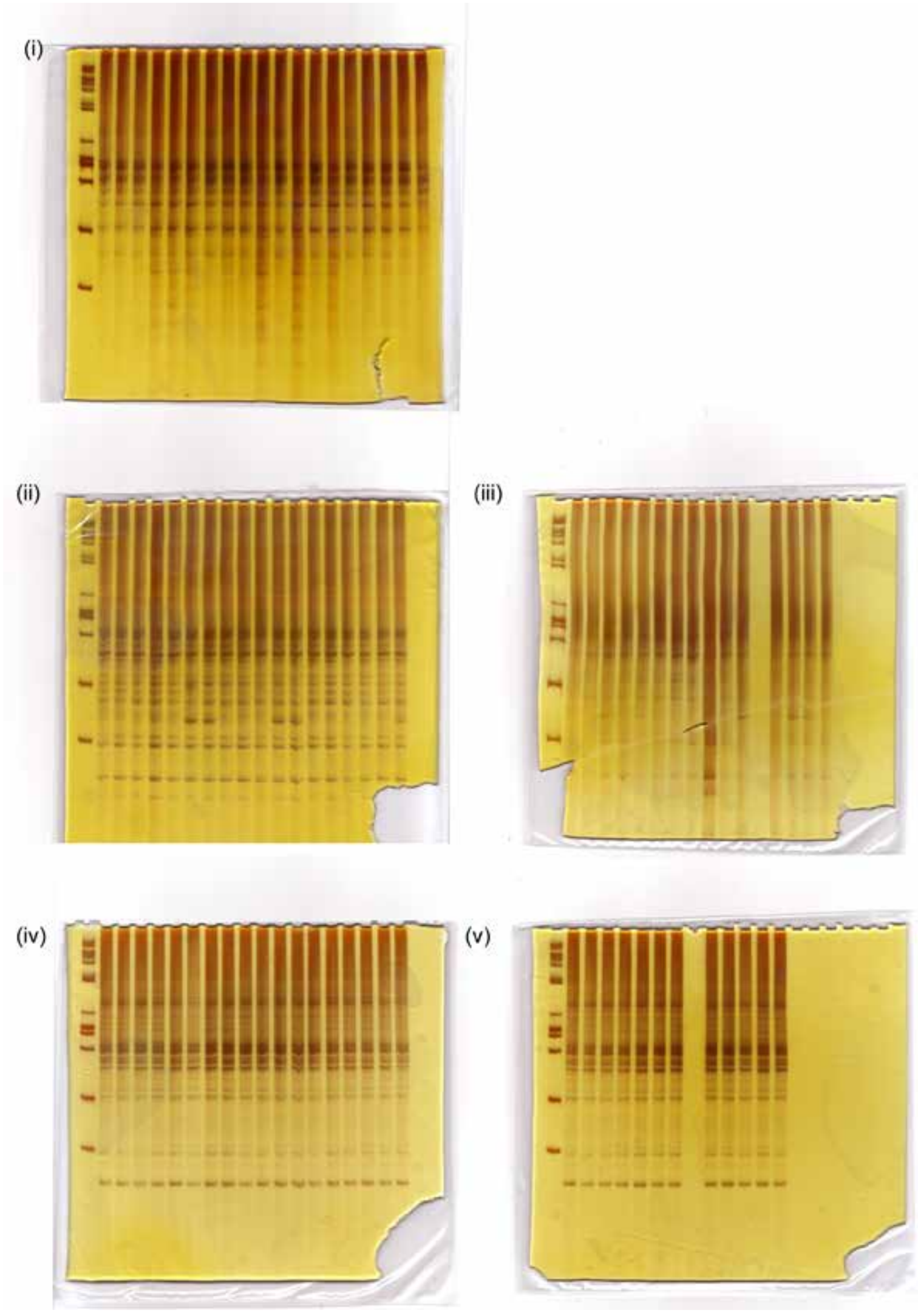

Figure K: PAGE gels from genotyping analysis of *Tubulipora plumosa*: within-brood comparison. Gel images show banding profiles of larvae (in triplicate) from each brood screened with the ISSR primer UBC 855: (i) Brood J, (ii-iii) Brood K, (iv-v) Brood L. Lane 1 = ladder. Note: Lane 13 in (iii) is blank due to PCR failure and Lane 9 in (v) is intentionally left blank.

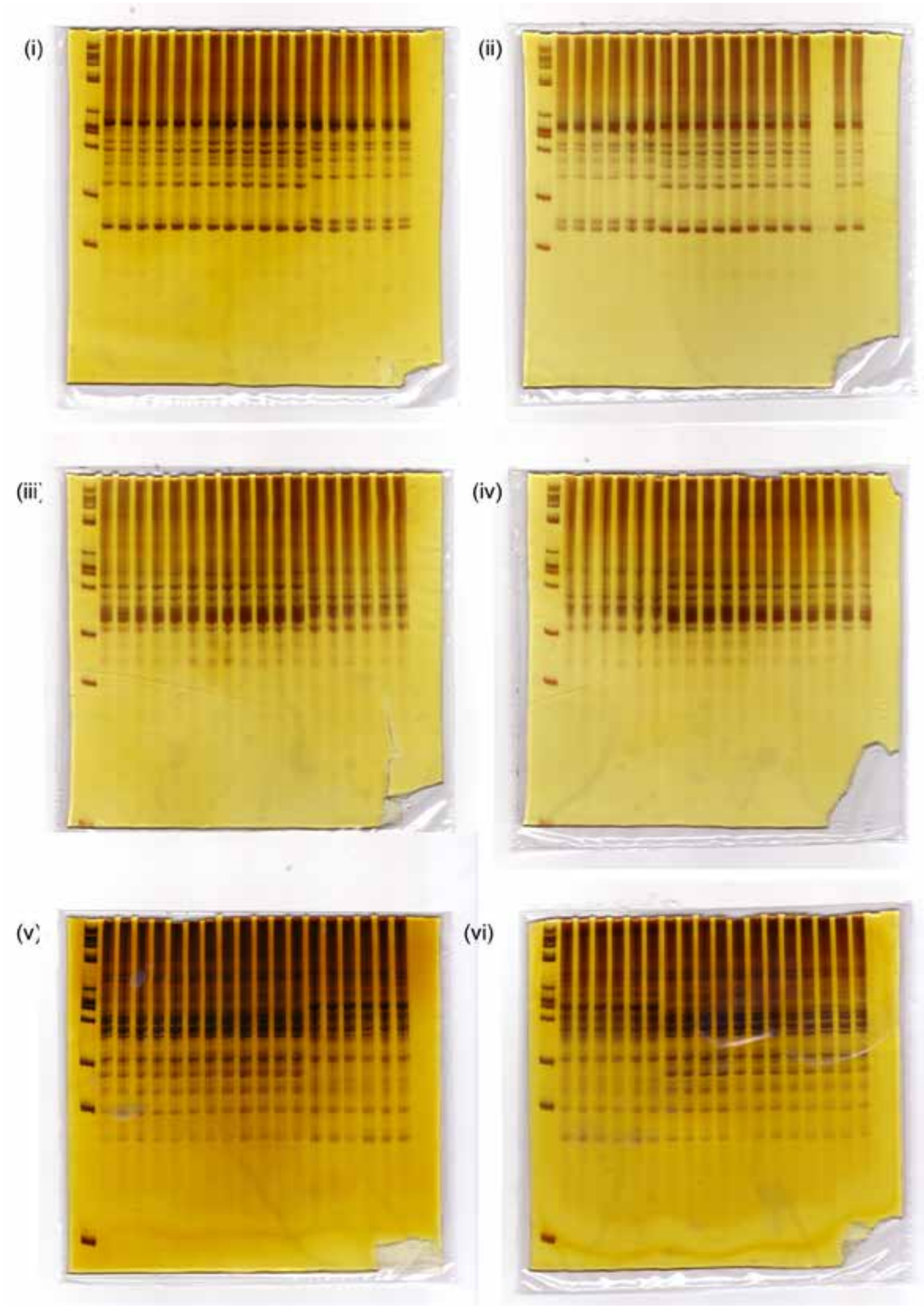

Figure L: PAGE gels from genotyping analysis of *Tubulipora plumosa*: within-brood comparison and comparison between broods within the same colony. Gel images show banding profiles of eight Brood M larvae and four tissue extracts from 'whole brood' sample WB 02 (all in triplicate) screened with the ISSR primers UBC 817, UBC 850 & UBC 855. UBC 817: (i) Brood M (lanes 2-13), WB 02 (lanes 14-19); (ii) WB 02 (lanes 2-7), Brood M (lanes 8-19). UBC 850: (iii) Brood M (lanes 2-13), WB 02 (lanes 14-19); (iv) WB 02 (lanes 2-7), Brood M (lanes 8-19). UBC 855: (v) Brood M (lanes 2-13), WB 02 (lanes 14-19); (vi) WB 02 (lanes 2-7), Brood M (lanes 8-19). Lane 1 = ladder. Note: Lane 17 in (ii) is blank due to PCR failure.
