# Supplementary material for: Phylogenetically Widespread Polyembryony in Cyclostome Bryozoans and the Protracted Asynchronous Release of Clonal Brood-Mates
Source: PLoS One. 2017 Jan 17;12(1):e0170010. doi: 10.1371/journal.pone.0170010 (PMC5240946; doi:10.1371/journal.pone.0170010)
Supplement: S2 Appendix — (PDF) [file pone.0170010.s005.pdf]

S2 Appendix. ISSR Genotyping Analysis – virtual gels

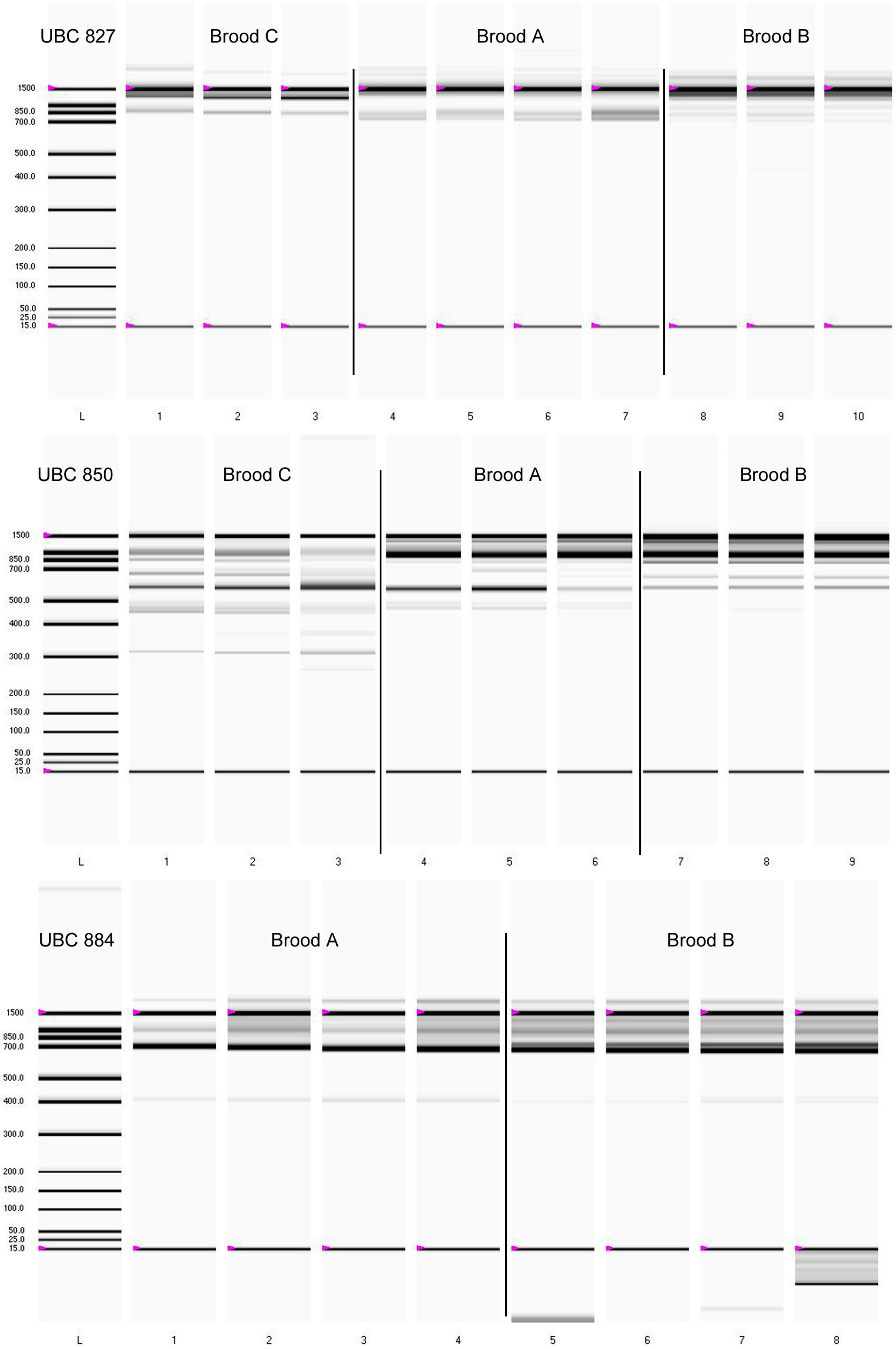

Figure A: Set of virtual gels from genotyping analysis of *Crisia denticulata*: comparisons between broods from different colonies. Each gel image shows banding profiles, from one ISSR primer, of three/four individuals (larvae) from one brood each from two or three different colonies. Note: Bands outside of the sizing range not scored. L = ladder. Upper internal size marker = 1500 bp, lower internal size marker = 15 bp.

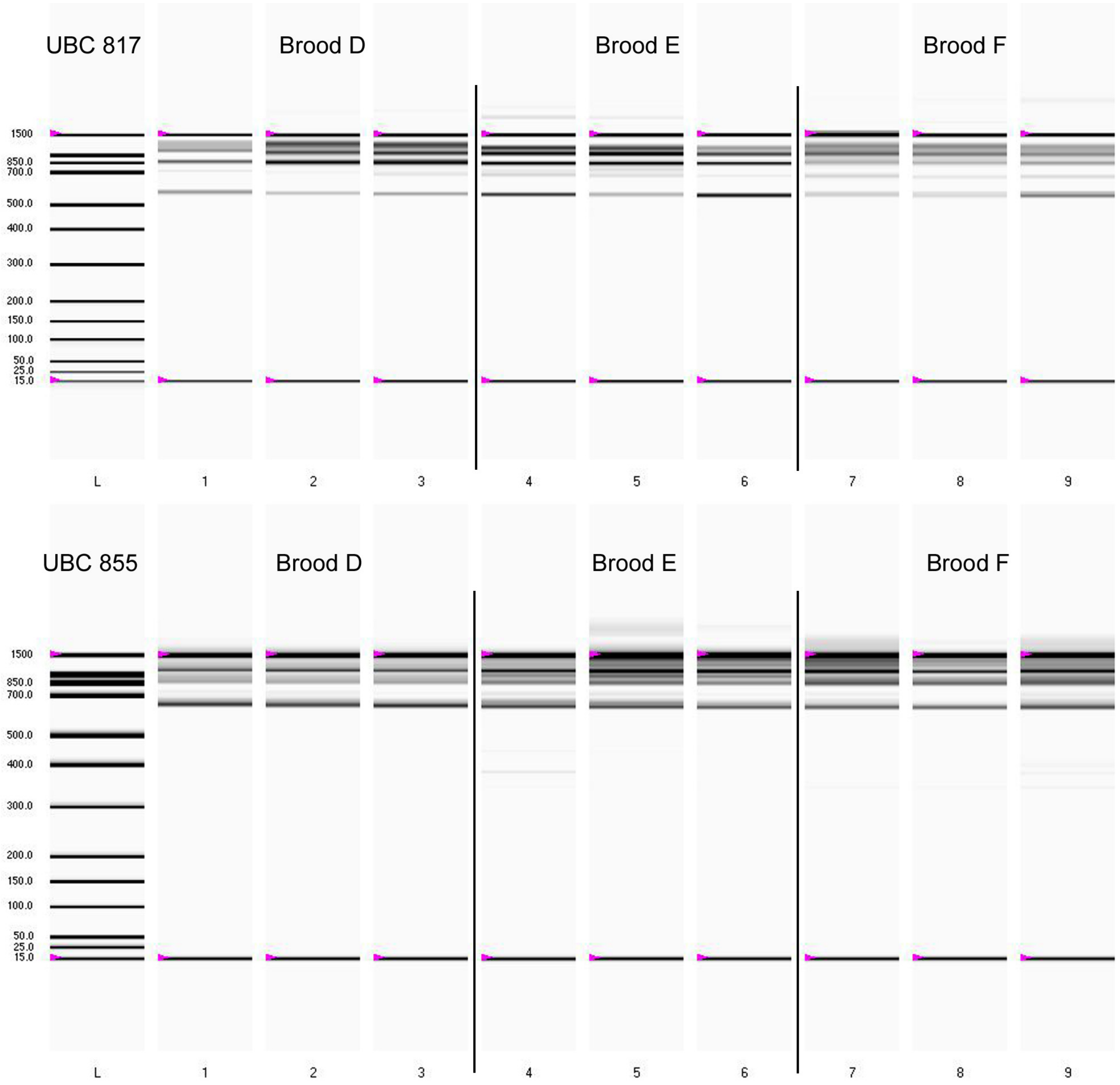

Figure B: Set of virtual gels from genotyping analysis of *Hornera robusta*: comparisons of broods from the same colony (Broods E & F) and different colonies. Each gel image shows banding profiles, from one ISSR primer, of three individuals (larvae) from one brood each from the same or two different colonies.
Note: Bands outside of the sizing range not scored. L = ladder. Upper internal size marker = 1500 bp, lower internal size marker = 15 bp.

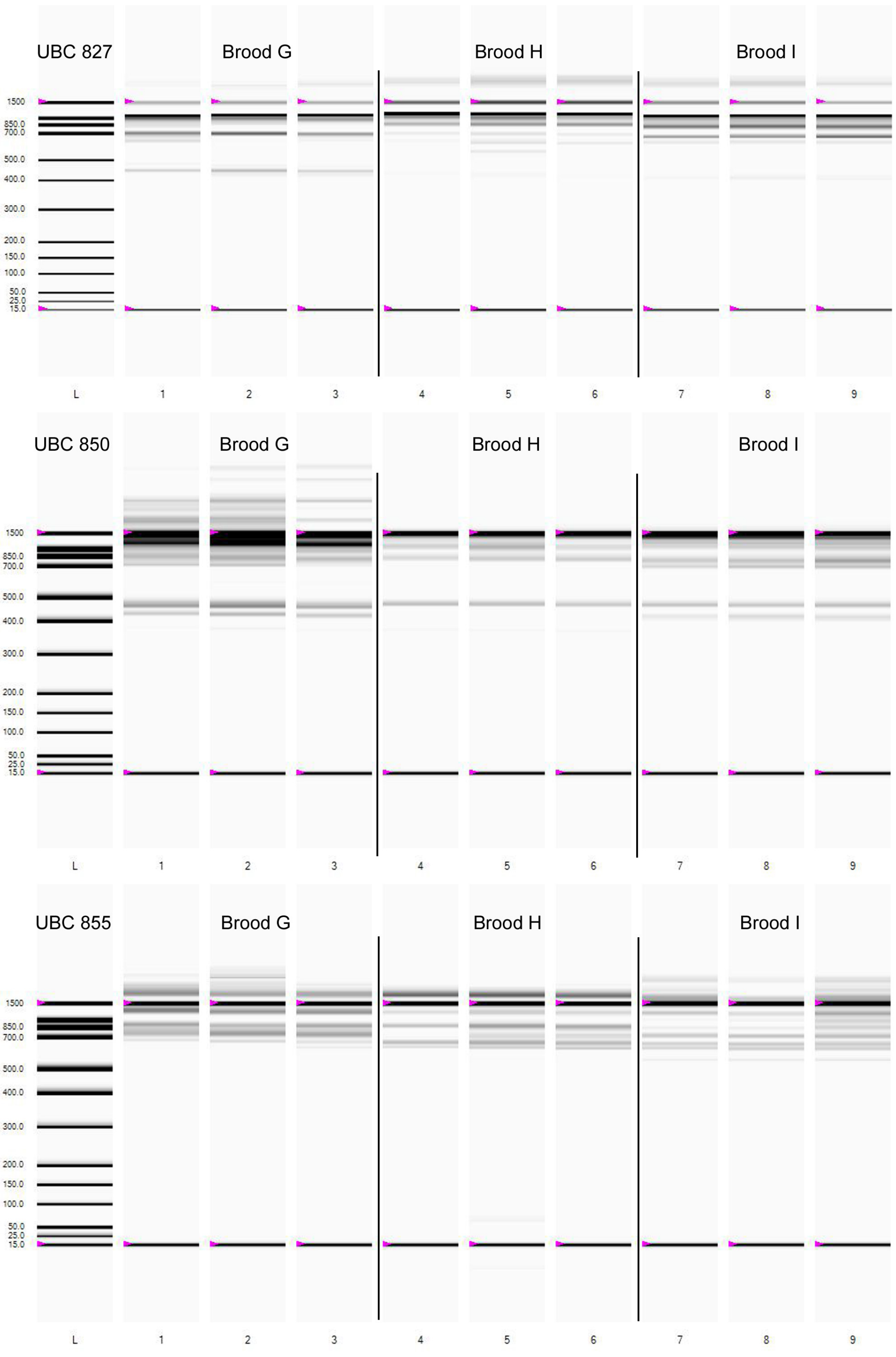

Figure C: Set of virtual gels from genotyping analysis of *Plagioecia patina*: comparisons between broods from different colonies. Each gel image shows banding profiles, from one ISSR primer, of three individuals (larvae) from one brood each from three different colonies. Note: Bands outside of the sizing range were not scored. L = ladder. Upper internal size marker = 1500 bp, lower internal size marker = 15 bp.

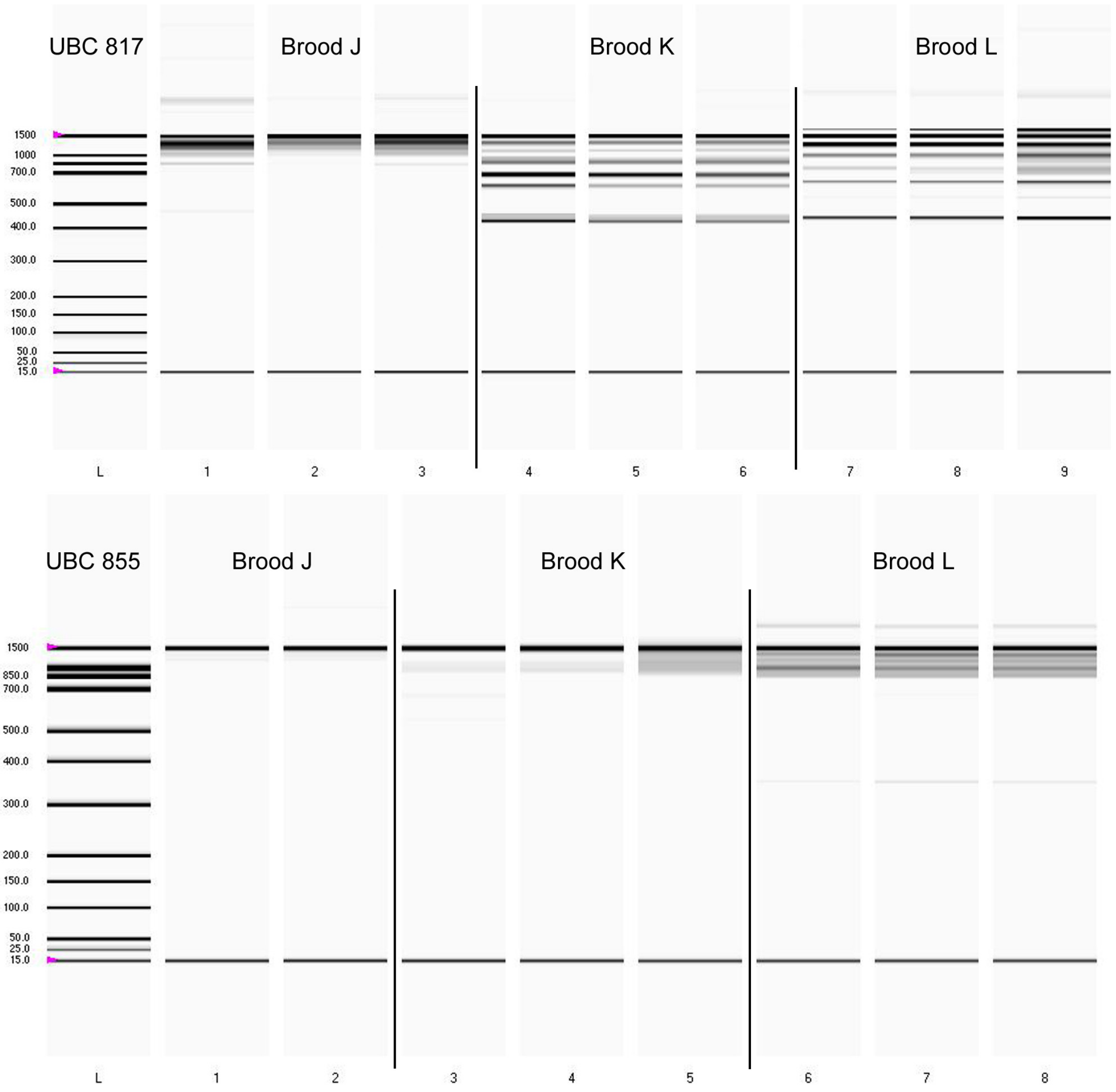

Figure D: Set of virtual gels from genotyping analysis of *Tubulipora plumosa*: comparisons between broods from different colonies. Each gel image shows banding profiles, from one ISSR primer, of two/three individuals (larvae) from one brood each from three different colonies. Note: Bands outside of the sizing range not scored. L = ladder. Upper internal size marker = 1500 bp, lower internal size marker = 15 bp.

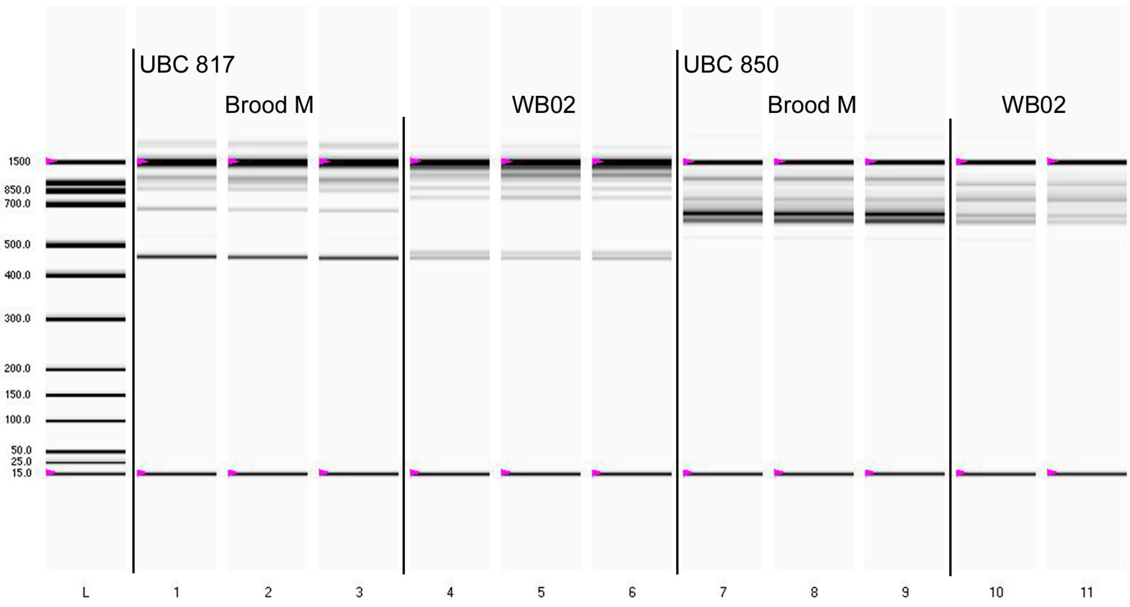

Figure E: Virtual gel from genotyping analysis of *Tubulipora plumosa*: comparisons between broods from the same colony. Gel image shows banding profiles, from two ISSR primers, for three individuals from Brood M and two-three different tissue extracts from the same brood WB02 ('whole brood' sample).
Note: Bands outside of the sizing range were not scored. L = ladder.
Upper internal size marker = 1500 bp, lower internal size marker = 15 bp.
